# Unveiling Tumorigenesis Mechanisms and Drug Therapy in Neuroblastoma by Mass Spectrometry Based Proteomics

**DOI:** 10.3390/children11111323

**Published:** 2024-10-30

**Authors:** Keyi Ren, Yu Wang, Minmin Zhang, Ting Tao, Zeyu Sun

**Affiliations:** 1 Department of Surgical Oncology, Children’s Hospital, Zhejiang University School of Medicine, National Clinical Research Center for Child Health, Hangzhou 310052, China; 2 Pediatric Cancer Research Center, National Clinical Research Center for Child Health, Hangzhou 310052, China; 3 State Key Laboratory for Diagnosis and Treatment of Infectious Diseases, National Clinical Research Center for Infectious Diseases, The First Affiliated Hospital, Zhejiang University School of Medicine, Hangzhou 310003, China; 4 Jinan Microecological Biomedicine Shandong Laboratory, Jinan 250118, China; 5 Key Laboratory of Diagnosis and Treatment of Neonatal Diseases of Zhejiang Province, Hangzhou 310052, China; 6 Cancer Center, Zhejiang University, Hangzhou 310058, China

**Keywords:** neuroblastoma, mass spectrometry-based proteomics, tumorigenesis, drug treatment, drug resistance, intratumor heterogeneity

## Abstract

Neuroblastoma (NB) is the most common type of extracranial solid tumors in children. Despite the advancements in treatment strategies over the past years, the overall survival rate in patients within the high-risk NB group remains less than 50%. Therefore, new treatment options are urgently needed for this group of patients. Compared with genomic aberrations, proteomic alterations are more dynamic and complex, as well as more directly related to pathological phenotypes and external perturbations such as environmental changes and drug treatments. This review focuses on specific examples of proteomics application in various fundamental aspects of NB research, including tumorigenesis, drug treatment, drug resistance, and highlights potential protein signatures and related signaling pathways with translational values for clinical practice. Moreover, emerging cutting-edge proteomic techniques, such as single cell and spatial proteomics, as well as mass spectrometry imaging, are discussed for their potentials to probe intratumor heterogeneity of NB.

## 1. Introduction

Neuroblastoma (NB) poses a significant challenge as the most common extracranial pediatric solid tumor, accounting for approximately 7–8% of all childhood malignancies and contributing to approximately 15% of childhood cancer-related mortality [1,2]. It originates from the embryonal sympathoadrenal lineage of the neural crest and can develop anywhere in the sympathetic nervous system, with the adrenal gland being the most common primary site [3,4]. Its heterogeneous nature leads to variable outcomes, from complete regression to aggressive metastasis despite multiple aggressive treatments [5,6]. Patients with low- and intermediate-risk NB have an excellent five-year survival rate of more than 90%. Surgery and reduced chemotherapy are typically provided for this group of patients. However, the five-year survival rate for high-risk NB patients, which account for approximately 60% of all NB cases, remains less than 50% [5,6,7]. Current treatments for high-risk NB include chemotherapy, surgical resection, high-dose chemotherapy with autologous stem cell transplant (ASCT), radiation therapy, immunotherapy, and isotretinoin treatment. Newly introduced trial treatments, such as anti-disialoganglioside (GD2) immunotherapy and 131I-meta-iodobenzylguanidine (131I-MIBG) therapy, has improved overall survival rates, but further advances in treatment are still needed [8,9]. Currently, NB diagnosis relies on a combination approaches of laboratory tests, radiographic imaging, and pathological examination, considering clinical risk factors such as tumor spread, age at diagnosis, and molecular risk factors including *MYCN* oncogene amplification, diploid DNA contents, and specific segmental chromosomal aberrations, such as 11q deletion, 1p deletion and 17q gain [3,10]. The International Neuroblastoma Risk Group Staging System (INRGSS) was developed to stratify NB patients into four stages (extremely low-risk, low-risk, intermediate-risk, and high-risk groups) for the NB pretreatment risk classification, based on clinical criteria and image-defined risk factors (IDRFs) [11,12].

Recent research has focused on exploring the molecular mechanisms involved in the NB pathogenesis. The investigation of genetic/protein aberrations and signaling pathways is critical for developing potential biomarkers and therapeutic targets for NB treatment [2]. For example, the amplification of *MYCN* oncogene has been associated with malignant progression, poor prognosis and reduced survival rates, which is detected in approximately 20–30% of all NBs [10,13]. Additionally, segmental chromosome alterations and genetic mutation, such as *ALK*, *PHOX2B*, *ATRX*, *PTPN11*, *ARID1A*, or RAS–MAPK and p53 signaling pathway alterations have been confirmed to be associated with worse outcomes and offer potential for precision therapy [9,14]. Compared with genomic aberrations, proteomic alterations are more dynamic and complex, as well as more directly related to pathological phenotypes and response to external perturbations such as environmental changes and drug treatments [15,16]. Some important protein signatures in NB and their related signaling pathways have been proposed as therapeutic targets, such as anaplastic lymphoma kinase (ALK), receptor tyrosine kinase (RTK), and aurora kinases A [17]. For example, alisertib as a regular aurora kinase A inhibitor, has been confirmed to enhance therapeutic effect with 131I-MIBG therapy in high-risk NB [18]. Another recent study illustrated that the suppression of DNA excision repair protein ERCC-6, also known as cockayne syndrome B (CSB), a pleiotropic protein and essential DNA repair factor, could hamper the proliferative, clonogenic, and invasive capabilities in NB, making CSB ablation a promising anticancer strategy for NB therapy [19]. Collectively, the exploration of novel protein targets holds a promising future for novel NB therapy development.

Transcriptomics (RNA-seq) techniques, with their capability for deep coverage and quantitation of low-abundance transcripts, have been widely applied nowadays [20]. However, transcript levels do not always correlate with protein abundance due to post-transcriptional editing and modifications. In contrast to transcriptomics, proteomics-particularly mass spectrometry (MS)-based proteomics-not only play a critical role in characterizing the proteome, assessing protein expression levels, and studying protein degradation and stability, but also offer direct insights into the functional protein landscape, including post-translational modifications, interactions, and localization, thereby revealing important signaling or metabolic pathways associated with biological or pathological processes [21,22]. Despite the widespread utilization of proteomics in various diseases, a comprehensive review of multifaceted applications of proteomics in NB research over the past five years is still lacking. To fill this gap, this review focuses on the application of MS-proteomics in NB studies of tumorigenesis, drug treatment and resistance, as well as other clinical aspects of NB. Furthermore, it discusses recent advancements in proteomics methodologies that may contribute to future studies on NB.

## 2. Advances in Mass Spectrometry-Based Proteomics Technologies

Proteomics employs protein microarrays, electrophoresis, and mass spectrometry. Although protein microarrays provide a straightforward workflow for large-scale quantitative proteomics, they are limited by the availability of specific and sensitive antibodies. Gel-based electrophoresis, particularly the two-dimensional electrophoresis (2DE), has long been used as a conventional protein separation method, but its low sensitivity and low throughput have restricted its application in contemporary biomedical research [23,24]. In contrast, the MS-based proteomic workflow, which typically analyzes thousands of peptides and proteins in a short time from various types of samples and species, significantly broaden the application scope of proteomics [25,26]. Over the past decade, advancements in sample processing, instrumentation, identification and quantification algorithms, and bioinformatics tools have propelled remarkable progress in MS-based proteomics (Table 1).

Two analytical procedures commonly used in MS-based proteomics are top-down and bottom-up approaches [26]. Bottom-up workflows account for majority of proteomic applications, which measure digested peptides as surrogates for the proteins of interest [27]. Most advanced quantitative proteomics methods are performed using bottom-up proteomics. Untargeted proteomics and targeted proteomics are two main types of data acquisition approaches in MS-based proteomics [28]. In most untargeted studies aimed at providing in-depth and unbiased analysis of the global proteome, the MS quantifies an enormous quantity of mixed peptides that have been pre-separated by liquid chromatography (LC), utilizing either in data-dependent acquisition (DDA) or data-independent acquisition (DIA) modes. In conventional DDA method, the eluted peptides from LC are firstly detected by full scan (MS1), and then a number of peptides are selected sequentially for identification by tandem mass spectrometry (MS/MS or MS2) [29]. As the prevalence of co-eluted peptide precursors usually exceeding the available MS2 spectra and the semi-stochastic selection of the precursor ions, the DDA method is often limited in the proteome coverage and reproducibility [30]. Therefore, DIA proteomics has received more attention in recent years, as it employs predetermined isolation windows including all co-eluding peptides for identification and quantitation, thereby achieving broader proteome coverage with high reproducibility and accuracy [31]. Traditional liquid chromatograph mass spectrometer (LC-MS/MS) based bottom-up proteomics determines peptides based on three dimensions, including LC retention time (RT), mass-charge ratio (*m*/*z*) and MS signal intensity. In recent years, the ion mobility was introduced as the fourth separation dimension for peptide ions. This four-dimensional (4D) proteomics greatly improves detection sensitivity with significantly enhanced separation resolution and identification depth [32]. Notably, 4D-proteomics combined with DIA technology, with its precursor ion sampling efficiency and higher precursor identification specificity, can comprehensively improve protein identification ability, detection sensitivity, and data integrity, thus attracting significant attention on contemporary proteomics studies [33,34].

Despite the advancements in DIA-based proteomics, traditional DDA approaches remain prevalent. Label-free quantification (LFQ) proteomics represents a cost-effective method for comparing protein expression across different samples; however, it exhibits relatively lower reproducibility [35]. For sample-wise multiplexing experiments, labeled approaches also have been developed. Stable isotope labeling by amino acids in cell culture (SILAC) is one of the common labeled approaches utilized for in vivo labeling in cell culture system, which achieve dynamic proteomic studies with comprehensive proteome coverage [36]. While for samples from clinical sources or animal models, in vitro labeling approaches such as isobaric tags for relative and absolute quantitation (iTRAQ) and tandem mass tags (TMT) can be employed with their high sample-multiplexing capacity and superior proteome coverage [37,38]. Post-translational modifications (PTMs) encompass phosphorylation, ubiquitination, glycosylation, acetylation, methylation, and others. PTM-proteomics, has become an essential field in understanding protein function and various cellular processes. Due to the low abundance, proteins or peptides containing PTMs usually require to be enriched prior to MS analysis. The PTM-proteomics usually have their suitable enrichment methods before MS/MS detection, including the use of specific antibodies, chemical probes, and affinity chromatography, to enhance the detection of low-abundance PTMs [39,40].

**Table 1:** Overview of the advanced MS-based proteomics techniques.

The Classifications on MS-Based Proteomics Techniques		Characteristics of Methods	Advantages
Data acquisition methods	DDA [29]	Selected numbers of fragmentation spectra of peptides are measured	Widely used, compatible with all quantification methods
	DIA [31]	All the precursor ions in the given range are acquired for the fragmentation	High proteome coverage, high reproducibility
	Targeted MS approaches [41]	Including selected reaction monitoring, multiple reaction monitoring and parallel reaction monitoring	Monitor the biologically important proteins and peptides in a complex mixture, high sensitivity
	4D-proteomics with DIA [33,34]	Ion mobility is introduced as the fourth separation dimension for peptide ions	High coverage, high reproducibility, capable of resolving PTM isoforms
Quantification methods	Label-free [35]	Measurement based on the intensity of peptide signals in MS without the use of labels	Cost-effective
	SILAC [36]	Culturing cells in isotope-containing culture media	High proteome coverage, high dynamic range and quantification accuracy
	iTRAQ/TMTs [37,38]	Add isobaric tags directly to enzyme-digested peptides	High proteome coverage, easy workflow with sample multiplexing
MS-based proteomics on PTMs [39,40]		Affinity enrichment process in pretreatment	Capable to explore subtle alteration in PTM level

DDA: data-dependent acquisition; DIA: data-independent acquisition; SILAC: stable isotope labeling by amino acids in cell culture; iTRAQ: isobaric tags for relative and absolute quantitation; TMT: tandem mass tags; PTM: post-translational modifications.

Despite the capabilities of MS-proteomics, the intricate complexity and multidimensionality of the proteome, along with the increasingly abundant datasets it generates, present a formidable analytical challenge for its widespread adoption. In addition to the consistent advancements in algorithmic solutions over the years, artificial intelligence (AI), particularly machine learning (ML) and deep learning (DL), have introduced a novel perspective for tackling this challenge [42]. For instance, DL models have been employed to predict peptide measurements from amino acid sequences, enhancing accuracy and reliability [43]; AI and ML play an indispensable role in biomarker discovery [44,45], outperforming traditional methods in identifying disease-related proteins. However, challenges such as overfitting issues, the black-box nature of DL models that lack transparent traceability [46], as well as privacy and data sharing concerns, necessitate resolution. Nonetheless, the application on integration of multi-omics data and transfer learning techniques driven by AI holds great potential for future breakthroughs [42,47].

## 3. Proteomics and Neuroblastoma

**Figure 1:**
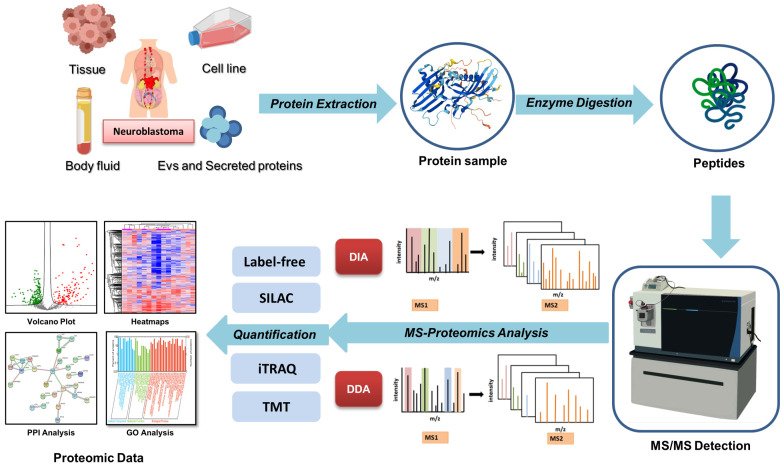
General Diagram of the workflow in proteomic studies in NB. Proteomic studies are applied to explore biomarkers and signaling pathways relevant to NB tumorigenesis, drug treatment and resistance, and they are carried out following with: (1) Collection of biological samples (tissues, cells, body fluid, EVs and other secreted proteins) from patients, animals, tissues or cell lines; (2) Collection of proteins by process of extraction and purification; (3) Collection of peptides after enzyme digestion; (4) MS/MS proteomics analysis with peptide samples based on different quantitative techniques: LFQ, TMT/iTRAQ, SILAC, DIA and others; (5) Data analysis with bioinformatics approaches, including expression difference analysis (exhibited with volcano plots, heatmaps and others), protein-protein interaction (PPI) analysis, gene ontology (GO) analysis, and additional approaches. EVs: extracellular vesicles; DDA: data-dependent acquisition; DIA: data-independent acquisition; SILAC: stable isotope labeling by amino acids in cell culture; iTRAQ: isobaric tags for relative and absolute quantitation; TMT: tandem mass tags; PPI: protein-protein interaction; GO: gene ontology.

The high-throughput proteomics techniques have been widely used to generate disease markers and unveil signaling pathways, which are related to critical biological processes and disease pathogenesis [48]. In this review, we provide an overview of the application of proteomics technologies in NB research. A literature search was conducted on the PubMed database from January 2019 to June 2024, using the search criteria encompassing the terms ‘neuroblastoma’ and ‘proteomics’. A total of 212 articles were retrieved, of which approximately 40 articles were relevant to the application of MS-based proteomics in NB. Only original research articles included while review articles, were excluded (2 articles). Articles utilizing NB cell lines as models for non-NB diseases, such as Parkinson’s disease or Alzheimer’s disease, were excluded (139 articles). Articles for NB study but without performing MS-based proteomic strategies during research were excluded (30 articles). These studies presented the advancements in proteomics approaches applied to NB, such as labeled-MS/MS methods and MS-based PTMs. The general workflows of MS-based quantitative proteomics in NB studies were outlined (Figure 1).

In this review, numerous significant protein signatures have been identified by MS-based proteomics over the past five years. Most of these surveyed literatures provided lists of significantly altered proteins and conducted notable analysis on these proteins’ mechanisms. Among these results, over 30 protein targets were emphasized, particularly those involved in established or potential mechanisms related to molecular risk factors and intricate molecular processes in NB. These protein findings significantly contributed to our current understanding of NB tumorigenesis mechanism, as detailed in Table 2. Furthermore, this review highlights the potential of proteomics technology in exploring drug treatment and resistance mechanisms in NB, which is shown in Table 3 and Table 4.

### 3.1. Proteomics Application in Neuroblastoma Tumorigenesis

NB is a complex and heterogeneous disease, with conventional risk stratification factors depending of the patient age, histological category, *MYCN* oncogene status, DNA ploidy, and specific segmental chromosomal aberrations, all of which demonstrate a strong correlation with molecular signatures in NB [3]. Genetic/proteomic aberrations such as *MYCN*, *BIRC5*, *PHOX2B*, and *LIN28B*, and molecular pathways alterations such as ALK signaling, MDM2, PI3K/Akt/mTOR and RAS-MAPK pathways, have potential to supplement traditional clinical risk stratification in NB [2]. Based on mechanisms associated with established molecular risk factors in NB, the proteomics technology has been extensively utilized in NB tumorigenesis investigation.

**Table 2:** NB study on tumorigenesis mechanisms identified by MS-proteomics and major discoveries in past five years.

Authors	Samples	MS-Proteomic Methods	Proteins Surveyed by Proteomics	Critical NB-Related Proteins Identified	Molecular Functions and Mechanisms
Cheng et al. [49]	SK-N-BE(2) and 293T cells	Label-free proteomics;	N-Myc interacting proteins	P300	Stabilizing N-Myc
Hsieh et al. [50]	Th-MYCN mouse model	iTRAQ-labeled proteomics	Proteins response to Aurora kinases inhibition	ACADM (+)	Inducing β-oxidation metabolism and reducing NB progression
Arlt et al. [51]	49 NB biopsies and 13 NB cell lines	Label-free proteomics;	Proteins correlated to *MYCN*-amplified level	PHGDH (+)	Inducing serine synthesis and one-carbon metabolism and promoting NB proliferation.
Yang et al. [52]	SK-N-BE(2), SK-N-DZ and SK-N-AS cells	-	lncRNA *SNHG1* interacting proteins	MATR3	Inducing RNA splicing and enhancing NB progression
Pedersen et al. [53]	SH-SY5Y cells	SILAC-labeled proteomics, TMT-labeled phosphoproteomics	Proteins response to Cbl proteins depletion	IGF1R (+), SHP2 (+), CDK16 (+)	Inducing ERK phosphorylation and promoting neurite outgrowth
Funke et al. [54].	IMR5 cell	Label-free proteomics; phosphoproteomics	Proteins response to NTRK1/TrkA activation	Lamin A/C/LMNA (+)	Inducing stability of nuclear lamina and NB differentiation
Emdal et al. [55]	NB1 cell	SILAC-labeled, TMT-labeled and label-free proteomics	ALK interacting proteins	IRS2	Stimulating PI3K-Akt-FoxO3 signaling and promoting NB cell survival
Uckun et al. [56]	SK-N-AS and SK-N-BE(2) cells	TMT-labeled proteomics	ALK interacting proteins	SHP2	Interacting with ALK and promoting NB proliferation
Li et al. [57]	Medium of SK-N-SH cell	-	Secreted proteins from S-type cells	PAI1, SPARC, POSTN and LEG1	Activating the STAT3 signaling and protecting cells from apoptosis
Hwang et al. [58]	SH-SY5Y cells	-	Proteins response to LGR5 knockdown	hnRNPH3, hnRNPA2B1 (−), and more	Activation of pre-mRNA processing and cell proliferation
Bugara et al. [59]	IMR-32 cells	-	Proteins response to *PHLDA1* activation	mitochondrion related proteins (+)	-
Dhamdhere et al. [60]	EVs derived from M1 and 9464D cells	TMT-labeled proteomics	Proteins correlated to IGF2BP1 level	SEMA3A (+), SHMT2 (+)	Inducing PMN formation and promoting metastasis of NB
Tsakaneli et al. [61]	EVs isolated from TET21-N NB cells	Label-free proteomics	Proteins correlated to *MYCN*-amplified level	PKM2 (+), hexokinase II/HK2 (+)	Enhancing histone H3 phosphorylation and promoting the metabolic activity in NB
Fonseka et al. [62].	Exosomes from SK-N-BE2 and SH-SY5Y cells	Label-free proteomics	Proteins correlated to *MYCN*-amplified level	Alix (+), TSG101 (+), FlOT1 (+) and VPS35 (+), and more	Regulating cell communication and signal transduction
Morini et al. [63]	Plasma exosomes from HR-NB patients and LR-NB patients and healthy controls	-	Proteins correlated to NB risk level	NCAM1 (+), NCL (+), LGALS3BP (+), LUM (−), VASP (−), DCN (−), MYH9 (+), FN1 (+), CALR (−), AKAP12 (−) and LTBP1 (+),and more	-
Garcia et al. [64].	SH-Y5Y cells	Label-free proteomics	Membrane proteins	NCAM1, L1CAM, EMMPRIN (CD147), ITGB1, ITGAV, CNTFR, and more	-
Gangras et al. [65].	SH-SY5Y cells	-	Membrane proteins	NRCAM	-

(+): Up-regulation; (−): Down-regulation; ACADM: medium-chain specific acyl-CoA dehydrogenase; PHGDH: phosphoglycerate dehydrogenase; RBPs: RNA binding proteins; IGF1R: Insulin-like growth factor 1 receptor; SHP2: SH2 domain-containing protein tyrosine phosphatase-2; Cbl proteins: casitas B-lineage lymphoma proteins; ALK: anaplastic lymphoma kinase; IRS2: insulin receptor substrate 2; PAI1: plasminogen activator inhibitor 1; SPARC: secreted protein acidic and cysteine rich; POSTN: periostin; LEG1: galectin-1; LGR5: leucine-rich repeat-containing G-protein coupled receptor 5; IGF2BP1: insulin-like growth factor 2 mRNA-binding protein 1; PMN: pre-metastatic niche; SEMA3A: semaphorin 3A; SHMT2: mitochondrial serine hydroxymethyl transferase 2; PKM2: pyruvate kinase M2; HK2: hexokinase II; NCL: nucleolin; LGALS3BP: galectin-3-binding protein; NCAM: neural cell adhesion molecule; LUM: lumican; VASP: vasodilator stimulated phosphoprotein; DCN: decorin; MYH9: myosin-9; FN1: fibronectin; LTBP1: latent-transforming growth factor-beta-binding protein 1; CALR: calreticulin; AKAP12: A-kinase anchor protein 12; NRCAM: neuroglia related cell adhesion molecule.

#### 3.1.1. MYCN-Related Mechanism

*MYCN* oncogene amplification has been integrated as a key risk factor into risk stratification and therapeutic strategies in NB research for several decades [10]. Its protein product N-Myc, as a transcription factor, was also associated with NB cell proliferation, invasion, angiogenesis and cell differentiation [66]. PTMs have been proven to play a vital role in regulating N-Myc oncoprotein, and corresponding regulatory enzymes could serve as potential targets for modulating N-Myc [67]. Cheng et al. performed endogenous and exogenous N-Myc coimmunoprecipitation (co-IP) followed by LC-MS/MS analysis in 293T cells and SK-N-BE(2) cells, to investigate the PTM residues and interaction proteins of N-Myc. Both acetylation and ubiquitination were identified on lysine 199 of N-Myc [49]. There were 14 potential N-Myc-interacting proteins identified, including several PTM regulatory enzymes. Subsequent investigation focused on histone acetyltransferase P300 as a potential N-Myc-interacting protein, which could acetylate N-Myc and regulate its ubiquitylation level. Further in vitro experiments confirmed that inhibition of P300 suppressed N-Myc level and correlated with favorable survival rates in NB.

Previous studies have shown aurora kinases A directly interacted with and stabilize N-Myc, leading to the NB development [68]. Hsieh et al. conducted iTRAQ-labeled quantitative proteomics in the Th-MYCN mouse model (*MYCN* overexpressed hemizygous mice with spontaneous NB) and identified 150 significantly differential expressed proteins, following treatment with the aurora kinase inhibitor tozasertib [50]. These proteins were mainly associated with metabolic processes, especially the carbohydrate and fatty acid metabolic pathways. Among these, the highly upregulated medium-chain specific acyl-CoA dehydrogenase (ACADM) was selected for further investigation due to its role in the β-oxidation metabolic pathway [69]. They further showed the high expression of ACADM was associated with a better survival in NB patients. Although the author suggested that inducing β-oxidation by inhibiting aurora kinases A may reduce NB progression mediated by ACADM, the exact mechanism and its relationship with N-Myc network still need to be clarified.

Arlt et al. employed LFQ proteomics analyses on 49 primary NB biopsies and 13 NB cell lines to investigate proteome alterations in various degrees of *MYCN* expression scores [51]. They identified the expression levels of 248 proteins in tumors and 38 proteins in cell lines were significantly correlated with *MYCN* expression. Spearman correlation analysis revealed that phosphoglycerate dehydrogenase (PHGDH) was positively correlated with *MYCN* level, exhibiting the highest affinity. The metabolic flux analyses and other existing databases demonstrated that PHGDH was involved in serine synthesis and one-carbon metabolism [70]. However, further functional experiments confirmed that *PHGDH* knockout and pharmaceutically inhibition slowed NB proliferation in the short term, but led to resistance to the standard treatment regimens. The chemoresistance was further verified in mouse models with patient-derived NB xenografts following treatment with PHGDH inhibitors and cisplatin.

Long non-coding RNA (lncRNA) *SNHG1* is significantly upregulated in NB, and is associated with poor patient prognosis and *MYCN* status [71]. However, the molecular mechanisms of *SNHG1* in NB are still unclear. Using RNA-protein pull-down assay combined with LC−MS/MS, 24 RNA binding proteins (RBPs) interacting with *SNHG1* were identified in all three NB cell lines (SKN-BE(2), SK-N-DZ and SK-N-AS) [52]. Among these proteins, DeepBind motif screening and co-expression analysis confirmed the high binding affinity between MATR3 and SNHG1 in all three cell lines. The gene set enrichment analysis (GSEA) and further studies confirmed the binding of MATR3 to *SNHG1* was involved in RNA splicing and cell cycle and was associated with poor patient survival. Although this paper provide a new clue that MATR3 could be a prognostic biomarker in high-risk NB, the more mechanism investigations on its relationship to *MYCN* network are still necessary in the future.

#### 3.1.2. RTK Signaling

RTK signaling has been found involved in mediating NB cell differentiation [72]. Although several key RTK signaling players have been documented, such as ALK and tropomyosin-related kinase A (TrkA), our understanding of the relationship between the RTK superfamily and NB tumorigenesis remains incomplete [73]. Signaling from RTKs is tightly regulated by E3 ubiquitin ligases, such as the casitas B-lineage lymphoma protein-Cbl proteins (Cbl/Cbl-b). Many previous studies found that depletion of Cbl and Cbl-b was associated with induction of neurite outgrowth [74]. To elucidate the global signaling network of Cbl proteins in NB, including its regulated proteins and PTMs, Pedersen et al. employed SILAC-based quantitative proteomics along with TMT-based phosphoproteomics to compare the protein alteration in Cbl/Cbl-b -depleted SH-SY5Y cells in response to various drug treatments [53]. Insulin-like growth factor 1 receptor (IGF1R), as the only RTK protein with significant upregulation in response to Cbl/Cbl-b depletion, was considered to be the target of Cbl/Cbl-b to induce NB cell differentiation. The following phosphoproteomics revealed that both phosphorylation and expression level of SH2 domain-containing protein tyrosine phosphatase-2 (SHP2) responded to Cbl/Cbl-b depletion. The SHP2, which linked to neurite outgrowth according to previous clinical trials [75], along with CDK16, a kinase involved with various signaling pathways [76], as well as IGF1R, were further validated in combination to induce ERK phosphorylation and neurite outgrowth mediated by Cbl protein-depletion.

High expression of the neurotrophin receptor NTRK1/TrkA is associated with favorable outcomes in NB. NTRK1 and its activation by nerve growth factor (NGF) have been shown to induce differentiation in NB [77]. To better understand *NTRK1* signaling and its link to *MYCN*, Funke et al. described global proteome and phosphoproteome alterations in IMR5 cells (a kind of *MYCN*-amplified cell line) under NGF treatment [54]. A total of 230 differentially regulated proteins and 147 significantly regulated phosphorylation sites were responsive to NTRK1 activation. Among these proteins, the nuclear laminar component Lamin A/C (LMNA) showed a consistent response to NTRK1 activation by NGF over a time-course. Moreover, the increased phosphorylation of LMNA-pS22 was identified as a prominent feature upon NTRK1 activation. Enrichment analysis and other in vitro experiments confirmed the functional relationship between stability of the nuclear lamina and *NTRK1* signaling. Although the authors provide new insights into the regulation of NB differentiation mediated by NTRK1 [54], the interdependence of LMNA, NTRK1 and *MYCN* deserve more investigation.

#### 3.1.3. ALK Signaling

ALK is a famous RTK in the insulin receptor superfamily, and its downstream signaling is related to the RAS/MAPK, PI3K/AKT, and JAK/STAT pathways [78]. *ALK* gene mutation is also a key genomic aberration, with high correlation to family history and recurrence of NB [79,80]. Oncogenic ALK is reported as druggable NB targets using tyrosine kinase inhibitors (TKIs) [81]. To comprehensively understand ALK signaling and its interactions in NB, Emdal et al. developed a novel integrated proximal proteomics strategy to simultaneously study the ALK interactome and phosphotyrosine interactome [55]. A list of 51 proteins that interacted with ALK was screened out with significant inhibition upon treatment with all three ALK-targeted TKIs. An additional 20 proteins were found directly bound to phosphorylated ALK tyrosine-containing peptides. In this newly constructed ALK interaction network, insulin receptor substrate 2 (IRS2) showing substantial changes in both expression level and tyrosine phosphorylation patterns, was proposed as a central node for ALK signaling transmission. The further abundant database analysis and functional experiments demonstrated the IRS2 as a proximal signaling adaptor promoting NB cell survival via PI3K-Akt-FoxO3 axis. Due to IRS2 being an adaptor protein that has been well characterized in the regulation of cellular glucose metabolism via insulin receptor (INSR) and IGF1R signaling [82], this paper may provide notable insight into the relationship between INSR/IGF1R and ALK signaling in NB.

In a similar study, Uckun et al. employed a biotin-based in vivo proximity labeling approach to identify intracellular partners of ALK [56]. In their study, ALK-BirA* (Escherichia coli biotin ligase) fusion protein were designed to be expressed in Tet-On NB cell lines (SK-N-AS and SK-N-BE(2)). ALK-concatenated BirA* can biotinylate proximal proteins, which can be purified by streptavidin and further analyzed by LC/MS-MS. They found 111 common candidates significantly enriched in ALK-BirA* NB cells compared to BirA* expressing cells. Many of these adaptor proteins have been previously reported to bind to ALK. The author further confirmed that SHP2 was an ALK interactor, and its interaction was enhanced by ALK ligand and was abrogated by lorlatinib (an ALK inhibitor). Further functional experiments confirmed that SHP2 could serve as a downstream target of ALK in NB cells and the combined inhibition of both ALK and SHP2 strongly decreases the proliferation of *ALK*-driven NB cells. The findings underscore the importance of proximal proteomics analysis in revealing ALK signaling networks, and these novel ALK interactors as potential therapeutic targets in *ALK*-driven NBs remain to be explored in future.

NB is highly heterogeneous and comprised of a mixture of neuroblastic cells and stromal cells. To test the sensitivity of these cell lines to ALK inhibition and related mechanism, the SK-N-SH cell line was developed as a cellular tool, which is composed of neuroblastic cells (N-type cells) and substrate-adherent cells (S-type cells), and both with *ALK* mutation in sequencing analysis [83]. To reveal the mechanism that secreted factors from S-type cell conditioned medium (CM) could protect N-type cells from apoptosis induced by the oncogenic *ALK* inhibitor TAE684., Li et al. used proteomics and identified the S-type CM harbored 74 specifically secreted proteins and most of them were related to biological adhesion [57]. Combined with RNA-Seq dataset and q-PCR verification, they selected the plasminogen activator inhibitor 1 (PAI1), secreted protein acidic and cysteine rich (SPARC), periostin (POSTN) and galectin-1 (LEG1) for further functional analysis. Notably, these four secreted factors were all able to activate STAT3 signaling via *ALK*-independent pathway, and combined inhibition on ALK and these significant factors could serve as a new direction for high-risk NB treatment.

#### 3.1.4. WNT/β-Catenin Signaling Pathway

WNT/β-catenin signaling has been found to be responsible for NB tumorigenesis [2], and the investigation in Wnt signaling may offer promising targets for therapeutic interventions in NB. Previous study has reported that the activation of leucine-rich repeat-containing G-protein coupled receptor 5 (LGR5) promotes Wnt/β-catenin signaling, and plays a critical role in NB cell proliferation [84]. MALDI-TOF-MS-based proteomics identified 12 protein spots altered by LGR5 knockdown in SH-SY5Y cells [58]. Among them, decreased protein expressions of hnRNPA2B1 and hnRNPH3 (hnRNP family) were verified by western blotting. According to this result and other in vivo/vitro experiments, the authors suggested a clue that the LGR5-Wnt/β-catenin signaling-mediated proliferation may be achieved via stimulation of hnRNPs in NB.

#### 3.1.5. Ganglioside GD2 Related Mechanism

Ganglioside GD2, is a glycolipid distributed on NB cell surface, and has been suggested as an immunotherapy target. Dinutuximab (ch14.18), a human/mouse chimeric monoclonal antibody (mAb) against GD2, has demonstrated efficacy to treat high-risk NB [85,86]. The anti-GD2 immunotherapy was proven to significantly increase survival of NB patients, but due to relatively high side effects and relapse, improvement is still needed for clinical implementation [87]. Proteomics profiling could offer comprehensive proteome alteration to investigate related mechanisms of anti-GD2 therapy. For instance, a recent study conducted a multi-dimensional proteomic, transcriptomic, and epigenetic analysis to evaluate GD2-targeting chimeric antigen receptor T cells (CAR-Ts) therapies in NB patient [88]. Additionally, considering that *PHLDA1* has been shown with significant activation upon anti-GD2 treatment in NB patients [89], Bugara et al. conducted a study on GD2 therapy in NB utilizing transcriptomic and proteomic analyses of IMR-32 cells. Their findings indicated that mitochondrion related proteome was significantly regulated by *PHLDA1* with the most pronounced alteration in expression level [59], thereby providing new protein therapeutic targets for combination strategies in GD2 immunotherapy.

#### 3.1.6. Extracellular Vesicles (EVs)

The contribution of small EVs to NB pathogenesis and resistance to therapies has been demonstrated in recent years [90]. EVs are a class of heterogeneously sized vesicles released by cells for intercellular communication, which including microvesicles (100 to 1000 nm) and exosomes (<200 nm) [90,91]. Recent studies have shown EVs could have a role as signaling factors that impact cancer progression by regulating the tumor microenvironment, immune responses, drug resistance, and oncogenic properties [92].

Previous studies have reported EVs could induce permissive metastatic microenvironment by regulation of pre-metastatic niche (PMN) formation in various cancers, including NB [93]. In the previous study by Dhamdhere et al., Insulin-like growth factor 2 mRNA-binding protein 1 (IGF2BP1) could regulate cell proliferation and impart chemoresistance to human NB cell lines in culture [94]. However, as IGF2BP1 did not exert any discernible impact on the cellular phenotype of the highly aggressive M1 cells (a newly established NB mouse cell line) in culture, it suggests its potential role through secreted factors such as EVs in NB. Using TMT-labeling proteomics to analyze the EVs from IGF2BP1-modulated cells (shIGF2BP1/shNT-M1 and IGF2BP1OE/EGFP control-9464D cells), the authors found the EV-enriched Semaphorin 3A (SEMA3A) and mitochondrial serine hydroxymethyl transferase 2 (SHMT2) were highly correlated with IGF2BP1 level [60]. Further in vivo validation demonstrated stimulation of IGF2BP1-SEMA3A/SHMT2 axis via EVs could promote PMN formation and NB metastasis.

Previous studies also showed that cancer cells expressing MYC proteins could modify the tumor microenvironment, possibly by regulating the secretion or content of EVs [95]. Tsakaneli et al. used LFQ proteomic analysis on EVs isolated from TET21-N cells with or without *MYCN* expression [61]. They found 111 upregulated and 41 downregulated proteins, primarily consisting of glycolytic enzymes, ribosomal proteins, and extracellular matrix (ECM) interaction proteins in the EVs derived from *MYCN*-positive cells. To focus on the relevant mechanism of glycolytic enzymes in the EVs, pyruvate kinase M2 (PKM2) and hexokinase II (HK2) were selected for further investigation. The PKM2-enriched EVs were confirmed to induce histone H3 phosphorylation and contribute to the aggressive behavior of NB by promoting the metabolic activity.

Exosomes are released by many cell types and implicated as key mediators in cell-cell communication via transferring their molecular cargo to the target cells [96]. Using LFQ proteomics targeting exosomes from SK-N-BE2 and SH-SY5Y cells with varying *MYCN* status (amplified versus non-amplified) and western blotting verification, Fonseka et al. revealed 581 proteins higher expressed in SK-N-BE2, such as Alix, TSG101, FlOT1 and VPS35. GO-based analysis highlighted the significant enrichment of proteins secreted by SK-N-BE2 cells in cell communication and signal transduction, as well as ErbB1, mTOR and integrin cell surface interaction signaling pathways, which suggested that *MYCN*-amplified cell-derived exosomes could regulate various signaling pathways in the recipient cells [62].

A latest similar study conducted a proteomics survey on isolated plasma exosomes from 24 high-risk (HR-NB) patients, 24 low-risk (LR-NB) patients and 24 age-matched healthy controls (CTRL) [63]. Upregulated proteins, such as nucleolin (NCL), galectin-3-binding protein (LGALS3BP) and neural cell adhesion molecule (NCAM), and the downregulated proteins, such as lumican (LUM), vasodilator stimulated phosphoprotein (VASP) and decorin (DCN) were found to be able to discriminate the NB patients from controls; meanwhile, up-regulation of myosin-9 (MYH9), fibronectin (FN1) and latent-transforming growth factor-beta-binding protein 1 (LTBP1), and down-regulation of calreticulin (CALR) and A-kinase anchor protein 12 (AKAP12) were also identified in HR-NB patients as compared to LR-NB patients. All of these interesting proteins were verified by q-PCR detection. These findings suggest that exosomes may play a crucial role in *MYCN*-driven aggressive NB.

#### 3.1.7. Membrane Proteomics

Comprehensive profilings of membrane proteins and their functions in NB have also gained increasing attention in recent years. The identification of prominent cell surface antigens in NB, such as glypican 2 (GPC2) and B7 homolog 3 (B7-H3, CD276), have paved the way for the advancement in novel targeted therapies for NB [97]. The initial investigation focusing on membrane proteins in NB employed cell surface biotinylation prior to LC-MS/MS analysis, and identified 2557 proteins in the cell surface-enriched fraction [64]. Based on label-free proteomic analyses, multiple cell surface proteins including NCAM1, L1CAM, EMMPRIN (CD147), ITGB1, ITGAV, and CNTFR, which were well characterized in other cancer types, were also considered as potential targets for NB treatment.

In a recent study conducted by Gangras et al., surface proteins were labeled on live SH-SY5Y cells with sulfo-NHS-SS-biotin for downstream enrichment prior to LC-MS/MS analysis [65]. By employing bioinformatic selection based on SurfaceGenie annotation [98] and GO cellular component terms, a total of 298 membrane-associated proteins expressed in the NB-derived SH-SY5Y cell were identified. Significant overlaps were observed between NB cell surfaceome and that of brain and dorsal root ganglion (DRG) neurons, but many protein isoforms were alternatively spliced in NB cells. Using RNA sequencing, the authors confirmed neuroglia related cell adhesion molecule (NRCAM) isoform 4 was absent in typical brain neurons but present in SH-SY5Y cell, making it a specific target candidate for NB therapeutics.

### 3.2. Proteomics Application in Drug Treatment

#### 3.2.1. Inhibitors of NB Molecular Risk Factors

Proteomic analysis offers valuable insights into changes in protein expression and signaling pathways modulated by drug treatments, thereby significantly advancing the discovery of drug targets and pharmaceutical mechanisms associated with NB. In current research in NB drug treatment, molecular inhibitors of prominent molecular risk factors, are often considered to have new drug development potential. However, the specific mechanisms and action targets of these small molecule drugs require thorough investigation in NB. MS-based proteomics has proven to be an effective tool for comprehensive proteome profiling in pharmaceutical research.

Ataxia telangiectasia and Rad3-related protein (ATR) is an essential kinase that activates cell cycle checkpoint signaling in response to DNA stress and damage and plays an important role in cancer cell survival [99,100]. Szydzik et al. combined RNA-Seq, proteomics and phosphoproteomic analysis data to investigate the effects of ATR inhibition with BAY 1895344 treatment on NB cells (CLB-BAR or CLB-GE) [101]. They validated the target proteins associated with E2F transcription factors, as well as several transcription factors known to be involved in DNA damage response, such as RAD51 and BRCA1/2. Furthermore, phosphoproteomic analysis identified 444 differentially expressed phosphorylated proteins in response to BAY 1895344 treatment and highlighted the ATR target proteins were significantly enriched in DNA repair machinery in NB cells, such as E2F3 and DCK. In vivo/vitro experiments further confirmed its potential to inhibit the growth of *ALK*-driven NB cells and xenograft, suggesting ATR inhibition could be a promising therapeutic strategy to treat NB, particularly for *ALK*/*MYCN*-driven NB patients.

Previous study has proved combined inhibition of ATR (with elimusertib) and ALK (with lorlatinib) could lead to a complete ablation of tumors in *ALK*-driven NB mouse models [101]. In another research on ATR inhibitor in NB, Borenas et al. compared phosphoproteomics profiling of CLB-BAR cells treated with either ceralasertib or elimusertib, two ATR inhibitors, and showed both drugs led to compensation of ATR inhibition by activation of ataxia-telangiectasia mutated (ATM) and DNA-dependent protein kinase (DNAPK) [102]. Moreover, phosphorylation sites in different PI3-kinase-related protein kinase (PIKK) family members (ATM, ATR, DNAPK, or mTOR) were further focused. This study confirmed that elimusertib or ceralasertib, which leads to a highly similar reduction of ATR and mTOR signaling, may be driven by a compensatory activation response of both ATM and DNAPK, thus showing a clue that combination therapeutic strategy based on ATR inhibitor for the treatment of *ALK*-positive NB patients.

Various small-molecule TKIs that target ALK are currently used in clinical treatment for both pediatric and adult patient populations [103]. However, the limited success of ALK inhibitor monotherapy raised requirements for further investigation on molecular mechanisms of TKIs-ALK [104]. Phosphoproteomic and RNA-seq analysis was applied to NB cell lines treated with either crizotinib or lorlatinib, the first- and third-generation ALK inhibitors, identifying many differentially phosphorylated components of the ALK signaling pathway [105]. This result provided multiple analyses on protein targets and signaling pathways upon TKI–treatment. Among them, MAPK phosphatase DUSP4 (also known as MKP2) was regulated at the level of both phosphorylation and transcription, and further studies showed that high DUSP4 abundance may enable the maintenance of a delicate ERK signaling balance, suggesting that DUSP4 might be a phosphatase involved in negative feedback for *ALK* signaling. This in-depth investigation of downstream targets of ALK signaling upon TKI–treatment offers future avenues for *ALK*-driven NB treatment.

*PI3K*/*Akt*/*mTOR* pathway has been identified as a viable treatment target for aggressive NB [106,107]. However, the intrinsic resistance and acquired resistance to PI3K inhibitors pose significant challenges to treatment efficacy in NB. Increased expression of serine/threonine proviral insertion sites in murine leukemia virus (PIM) kinases has been associated with PI3K inhibitor resistance [108,109]. Mohlin et al. synthesized IBL-302, as a novel highly specific triple PIM, PI3K, and mTOR inhibitor, with the aim of enhancing the NB treatment efficacy [110]. Global RNAseq, proteome, and phosphoproteome analyses on PDX-derived cells revealed several cellular processes were influenced by IBL-302 treatment, including cell apoptosis, programmed cell death, and cell cycle. Notably, caspase-3 and CDK6 were the most significantly differentially upregulated proteins. The multi-omics analyses and sufficient in vivo/vitro experiments shed light on *PIM*/*PI3K*/*mTOR* inhibition as a promising combinatorial therapeutic strategy to improve clinical outcomes in NB.

Mitochondrial division inhibitor 1 (Mdivi-1) is a well-known synthetic compound that disrupts mitochondrial dynamics by targeting dynamin-related protein 1 (Drp1), and is reported to induce programmed cell death in many cancers [111,112]. Wang et al. utilized formaldehyde-H2 or formaldehyde-D2-labeled proteomic and phosphoproteomic analysis to provide comprehensive insight into the biological processes induced by Mdivi-1 in the SK-N-BE(2) [113]. Among the significant modulated proteins, three enzymes essential to serine synthesis were found to be activated upon Mdivi-1 treatment, namely PHGDH, PSAT1, and PSPH. The dephosphorylation of PSMA3-S250 was also focused on in this research with its function of curtailing proliferation.

MiR-204 is known as a tumor suppressor that targets multiple oncogenes, including *MYCN*, *BCL2*, *NTRK2* and *PHOX2B*, which are associated with tumor progression and chemoresistance in NB [114,115,116]. However, the administration of miRNA mimics with liposomal nanoparticles as carriers may induce serious immune-mediated side effects in body [117]. Extracellular nanoparticles produced from red blood cells (REPs) can be used as biocompatible nanocarriers for RNA drug delivery [118]. To obtain mechanistic insights of miR-204-loaded REPs (REP-204) therapeutic effects on NB, Chiangjong et al. utilized SWATH-proteomics and bioinformatics analysis on REP-204 treated SK-N-BE2 and SH-SY5Y cells, comparing them to non-treatment conditions [119]. Based on their proteomic data and existing reports, interference with mRNA splicing machinery and SLIT/ROBO pathway were considered as main mechanisms upon REP-204 treatment. This study provided more evidence of anti-NB activity of REP-204, but further verification in in vivo model is warranted.

The nuclear-to-cytoplasmic transport protein Exportin-1 (XPO1) is an essential regulator for nuclear cargo proteins export [120], and is associated with poor prognosis in various adult cancers. Galinski et al. conducted LFQ based proteomic comparison on tumors from 50 patients and demonstrated that XPO1 was one of the most highly abundant proteins associated with the most aggressive diseases, suggesting that XPO1 is a potential prognostic biomarker and therapeutic target in NB [121]. Selinexor is a first class small molecule inhibitor of XPO1, which has shown therapeutic benefits in some adult cancers [122]. In Nguyen et al.’s study, TMT labeling based proteomics and phosphoproteomic analyses were conducted to identify critical target proteins and pathways influenced by selinexor [123]. NB cell line (KCNR, SH-SY5Y) and two *MYCN*-amplified patient-derived xenografts (PDX) lines with selinexor exposure were tested, and 11,174 unique proteins and 46,755 phosphopeptides were screened. The data revealed prominent increase in p53 protein expression. And the increased phosphorylation at site S315 in response to selinexor exposure was known as the initiating step for p53 degradation [124]. Combined with more in vivo/vitro experiments, it revealed a potential therapeutic benefit using selinexor to increase p53-mediated cytotoxicity in high-risk NB.

The MAPK pathway activity can lead to cell proliferation and tumorigenesis in NB. ERK is a major effector kinase in the MAPK pathway that activates various substrates through phosphorylation, possibly contributing to the chemoresistance and recurrence rate in NB [125,126]. In the work conducted by Yu et al., proteomic analysis was performed on NGP cells treated with ulixertinib, an ERK inhibitor [127]. A list of 72 upregulated and 85 downregulated proteins with ulixertinib treatment were found, which significantly related to the inhibition of cell cycle-related pathways and DNA replication/synthesis pathways. Among these proteins, ulixertinib enhanced the level of pro-apoptotic effector PRUNE2 [128] and cellular proliferation marker MK167 [129], providing new insights into the working mechanism of ulixertinib in NB. However, the author only listed the altered proteins identified by proteomics in this research, without employing enough functional experiments to investigate the mechanisms involving these specific targets with ulixertinib.

**Table 3:** NB study on drug therapy identified by MS-proteomics and major discoveries in past five years.

Authors	Samples	MS-Proteomic Methods	Drug Name	Information of Drug	Protein Targets and the Response to Drug	Related Mechanisms of Drug
Szydzik et al. [101]	CLB-BAR and CLB-GE cells	TMT-labeled proteomics, phosphoproteomics	BAY 1895344	ATR inhibitor	RAD51 (−), BRCA1/2 (−), E2F3 (−), DCK (−) and more	Inhibiting E2F transcription and DNA repair machinery pathways
Borenas et al. [102].	CLB-BAR cells	Phosphoproteomics	Ceralasertib, Elimusertib	ATR inhibitor	phospho-ATM (+),phospho-DNAPK (+), and more	Decreasing ATR and mTOR signaling
Van et al. [105]	CLB-BAR, CLB-GE, SK-N-AS cells	Label-free phosphoproteomics	Crizotinib or lorlatinib	ALK inhibitor	DUSP4 (−) and more	Inducing feedback loop of ERK and ALK signaling
Mohlin et al. [110]	LU-NB-3 cells	Label-free proteomics, phosphoproteomics	IBL-302	PIM, PI3K, mTOR inhibitor	Caspase3 (+) and CDK6 (+), and more	Inducing programmed cell death and cell cycle signaling
Wang et al. [113].	SK-N-BE(2) cells	formaldehyde-H2 and formaldehyde-D2-labled proteomics and phosphoproteomics	Mdivi-1	Mitochondrial division inhibitor	PHGDH (+), PSAT1 (+), PSPH (+), PSMA3 (−), and more	Inducing serine synthesis, curtailing proliferation, and more
Chiangjong et al. [119]	SK-N-BE2 and SH-SY5Y cells	-	miR-204-loaded REPs	MiR-204 mimic	-	Suppressing mRNA splicing and SLIT/ROBO pathway
Nguyen et al. [123]	KCNR, SH-SY5Y cell lines and PDXs	TMT-labeled proteomics, phosphoproteomics	Selinexor	XPO1 inhibitor	P53 (+), and more	Increasing p53-mediated cytotoxicity
Yu et al. [127]	NGP cells	Label-free proteomics	Ulixertinib	ERK inhibitor	MK167 (+), PRUNE2 (+), and more	Inhibiting Cycle-related and DNA replication/synthesis pathways
Chandel et al. [130]	SH-SY5Y cells	Label-free proteomics	EAD	Limonoid	ENO1 (−) and HSP90 (−),and more	Preventing proliferation and triggering apoptosis in NB
Laghezza et al. [131]	SH-SY5Y cells	Label-free proteomics	HTyr-OL	Polyphenol	BAG3 (+), HMOX1 (+), CLU (+), HERPUD1 (+), and more	Inducing apoptotic signaling pathway
Forbes et al. [132].	SH-SY5Y, IMR-32, BE(2)-C, GI-M-EN, SK-N-AS cells	LFQ proteomics, phosphoproteomic	L-Glyceraldehyde	Monosaccharide	PARVA (+), TP53 (+), DTD1 (+), and more	Increasing oxidoreductase activity and inhibiting cell growth
Lee et al. [133]	SH-SY5Y cells	Label-free proteomics	MS13	Curcumin analog	ENO1 (−), HSP90AA1 (+), HSP90AB1 (+), TUBB (−), and more	Inducing glycolysis and PTM-modification pathways
Morretta et al. [134]	HTLA-230 cells	Label-free proteomics	STIRUR 41	Pyrazolyl-urea and dihydro-imidazo-pyrazolyl-urea compounds	USP-7 (−)	-
Chittavanich et al. [135]	RB organoids	Label-free proteomics	Ceftriaxone	Third generation cephalosporin antibiotic for MYCN-driven tumors’ inhibition	DDX3X (−)	Inhibiting *MYCN* translation
Halakos et al. [136]	SK-N-SH cells	Label-free proteomics	13-cis RA	Vitamin A derivative used in the clinic post-chemotherapy	ICAM1 (+), NEFM (+), CRABP2 (+), PLAT (+) and more	Reducing ECM and collagen metabolic process, and promoting neurofilament formation
Halakos et al. [137]	SK-N-SH cells	Label-free proteomics	K777 and 13-cis RA	K777: cathepsin inhibitor	ARHGEF2 (+), B2M (+), CRABP2 (+), NEFL (+), TCF12 (+), APP protein family (+), and more	Inducing neuronal differentiation

(+): Up-regulation; (−): Down-regulation; ATR: Ataxia telangiectasia and Rad3-related protein; ALK: anaplastic lymphoma kinase; XPO1: exportin-1; ATM: ataxia-telangiectasia mutated; DNAPK: DNA-dependent protein kinase; Mdivi-1: mitochondrial division inhibitor 1; EAD: epoxyazadiradionex; ENO1: enolase1; HTyr-OL: hydroxytyrosyl oleate; TUBB: tubulin beta chainx; USP-7: ubiquitin carboxyl-terminal hydrolase 7; RB: retinoblastoma; 13-cis RA: 13-cis retinoic acid; The table and corresponding text is organized in alphabetical order of the drug names during each section.

#### 3.2.2. Natural or Synthesis Compounds for NB Treatment

Animal or plant-derived compounds have long been explored for their potential antitumor activities, yet the specific targets and action mechanisms in NB remain largely unknown. Epoxyazadiradione (EAD) is a limonoid, derived from Azadirachta indica [138], with anti-cancer potential against NB. To identify the specific EAD target, a LFQ proteomic approach was conducted on SH-SY5Y cells with or without treatment of EAD [130]. This study revealed that the expression of Enolase1 and HSP90 decreased in a concentration-dependent manner upon treatment with EAD. In vitro experiments further confirmed that EAD can prevent proliferation and trigger apoptosis in NB cells via Enolase1 and HSP90 pathways.

Hydroxytyrosyl oleate (HTyr-OL) has been structural optimized from a polyphenol, hydroxytyrosol (HTyr), derived from extra virgin olive oil, with high lipophilicity and bioavailability. By utilizing LFQ proteomic approach, Laghezza et al. identified 220 unique proteins in NB cells (SH-SY5Y) that responded to HTyr-OL treatment [131]. Among these upregulated proteins, BAG family molecular chaperone regulator 3 (BAG3) has been reported to be implicated in the extrinsic apoptotic signaling pathway, while Heme oxygenase 1 (HMOX1), Clusterin (CLU), and Homocysteine-responsive endoplasmic reticulum-resident ubiquitin-like domain member 1 protein (HERPUD1) are connected to the intrinsic apoptotic signaling pathway triggered by DNA damage. These proteins can be considered as the downstream targets of HTyr-OL treatment for further research.

Glyceraldehyde (GA) is a three-carbon monosaccharide, was reported to inhibit glycolysis and cell growth [139]. Previously published experiment has shown that NB cells were sensitive to GA in vivo, with inhibited glycolysis and cell proliferation [140]. Forbes et al. performed LFQ proteomic analysis of L-GA treated NB cells (SH-SY5Y, IMR-32, BE(2)-C, GI-M-EN, SK-N-AS), and protein signatures associated with oxidoreductase activity were focused [132]. Combined with further phosphoproteomic results, PARVA, TP53, DTD1, MAPT, and CDC25B were regarded with the strongest response following L-GA treatment. However, limitations in in vivo/vitro verification make it difficult to identify the actual molecular functions induced by L-GA in NB.

In the study by Lee et al., the pharmacological mechanism of curcumin analog MS13 was investigated in human glioblastoma U-87 MG and NB SH-SY5Y cells using LFQ proteomic analysis [133]. In both cells, exposure to MS13 resulted in significantly altered expression of heat shock protein HSP 90-alpha (HSP90AA1, HSP90AB1), tubulin beta chain (TUBB), and alphaenolase (ENO1). Reactome pathway database analysis showed “Glycolysis” and “Post-translational protein modification” were the two common pathways identified in both cells. Conclusively, MS13 demonstrates an anti-cancer effect that may indicate its potential use in the management of NB and brain malignancies.

Pyrazolyl-urea and dihydro-imidazo-pyrazolyl-urea compounds (STIRUR 13, STIRUR 41 and BUR 12) have been demonstrated to exert a potent inhibitory effect on interleukin 8 induced chemotaxis of human neutrophils. STIRUR 41 was also considered with ability to inhibit high-risk-NB recurrence, blocking inflammation and angiogenesis in NB [141]. Morretta et al. applied optimized drug affinity-responsive target stability (DARTS) based proteomics using in situ digestion and LC-MS/MS experiments [142,143] to investigate the effects of STIRUR 41 on HTLA-230, a stage-IV NB cell line with *MYCN* amplification [134]. Several putative targets from different biological pathways were identified, including ubiquitin carboxyl-terminal hydrolase 7 (USP-7), nuclear RNA export factor 1 (NXF1), and pescadillo homolog (PES1). USP-7 was chosen for further analysis due to its strong affinity for STIRUR 41, as confirmed by immunoblotting. The subsequent experiments demonstrated that treatment with STIRUR 41 could inhibit the both expression level and enzymatic activity of USP-7. In this research, authors conducted extensive analysis on the affinity of USP-7 to STIRUR 41, to verify USP-7 as a potential target, but did not further investigate the related mechanisms involved in NB.

#### 3.2.3. Drug Repurposing

Drug repurposing presents an opportunity to develop existing drug molecules for new therapeutic indications [144]. Some clinical drugs that have been performed in other diseases have shown potential for treating NB and warrant further investigation regarding their function and mechanism. Ceftriaxone, an FDA-approved third generation cephalosporin antibiotic, has been demonstrated with the ability to reduce the volume of unexpected retinoblastoma (RB) with *MYCN* amplification [135,145]. Chittavanich et al. developed a novel therapeutic tool—drug target identification (DTI), which combines affinity-based proteomics and molecular docking approaches, to illustrate action mechanism of ceftriaxone [135]. The GO analysis showed enrichment of “nucleic acid/RNA/mRNA metabolic process”, and the RNA helicases DDX3X was considered as a potential target of ceftriaxone. Ceftriaxone-DDX3X binding was also confirmed by western blotting. Their further study revealed that ceftriaxone may repress *MYCN* translation via targeting DDX3X. Thereby, ceftriaxone holds promise as a novel strategy for treating *MYCN* amplified NB in high unmet needs.

Furthermore, deep investigation into downstream targets of existing NB clinical drugs contributes to the rational design of new indications. 13-cis retinoic acid (13-cis RA), is commonly used in the clinic post-chemotherapy due to its differentiating effects, and also has been used in the NB clinical treatment [146]. Halakos et al. used LFQ proteomics analysis in SK-N-SH cells treated with 13-cis RA and identified significantly altered proteins primarily involved in reduced extracellular matrix synthesis (ECM) and collagen metabolic process, as well as increased neurofilament formation, all contributing to development and/or differentiation in NB [136]. Nuclear transporter-CRABP2, adhesion protein-ICAM1 and cytoskeleton protein-NEFM were upregulated, which were considered as important markers of neuronal differentiation. Notably, PLAT, which facilitates neurite outgrowth [147], was also upregulated in 13-cis RA treated cells. This proteomic investigation offered an extensive overview of the proteomic network influenced by 13-cis RA and identified potential targets for more effective treatment of NB.

The use of 13-cis RA has improved outcomes in NB, but relapse is still common in many high-risk patients with multiple aberrant genes. Thereby, drug combinations targeting different aberrant pathways simultaneously were recommended to improve the treatment efficacy and reduce relapse [148,149]. Irreversible cathepsin inhibitor K777 could induce autophagy and reduce tumor volume in NB [150]. Halakos et al. explored the potential of combining 13-cis RA with a cathepsin inhibitor (K777) to enhance the therapeutic efficacy of NB [137]. LFQ proteomics was employed to identify proteins affected by treatment with K777, 13-cis RA, and their combination in SK-N-SH cells, respectively. Multiple analyses comparing these sample groups provided several key protein signatures, which were validated by western blotting. In the result, when K777 was combined with 13-cis RA, ARHGEF2, B2M, CRABP2, NEFL, and TCF12 were increased, suggesting the dual treatment provides a more robust effect on neuronal development; Additionally, the APP protein family, which exhibited the most significant differential alterations identified through proteomic analysis, demonstrated that the combined treatments synergistically enhanced neuronal differentiation in NB.

#### 3.2.4. Proteomic Investigation of Drug Resistance

In current research in NB drug treatment, it is common for high-risk NB patients to develop drug resistance during chemotherapy, even leading to relapse [3]. Therefore, exploring more effective targets that can reduce drug resistance has been the focus in NB research for decades [151]. The application of MS-based proteomic analysis in NB provides deep insights into the molecular underpinnings of resistance mechanisms, which are essential for developing more effective treatments and improving the prognosis for NB patients.

In Wang et al.’s study, SILAC labeling based proteomics was used to compare two stable cell lines with different drug sensitivities [152]. SK-N-BE(1) and SK-N-BE(2) cells were isolated from the bone marrow of the same 2-year-old patient before and after several courses of chemotherapy, respectively. Among more than 460 significantly regulated proteins, Annexin A2 (ANXA2) was highlighted for over 12-fold upregulation in the chemoresistant NB cell line. Both in vitro and in vivo experiments revealed that ANXA2 inhibition induced more apoptosis with chemotherapeutic drugs and attenuated transcriptional activity of *NF-κB*, a key factor related to drug-resistance in NB [153]. These results suggested ANXA2 could be a prognostic biomarker and therapeutic target for multidrug-resistant NB patients. Furthermore, the authors presented an additional proteome dataset associated with drug resistance that may serve as potential targets for future investigation.

In another similar research, Tang et al. performed TMT-labeled quantitative proteomic analysis on NB tumor samples, comparing patients with a favorable prognosis to those with an unfavorable prognosis [154]. The result showed proteins involved in alternative splicing pathway, particularly the polypyrimidine tract binding protein 2 (PTBP2), were the most significantly upregulated in NB patients with favorable prognosis, which was also validated at mRNA level. Public microarray datasets showed the expression of PTBP2 was lower in NBs with relapse than in those without relapse. Further functional studies verified that PTBP2 induced the chemotactic activity and repolarization of tumor-associated monocytes/Mϕs in NB cells, thereby inhibiting tumor growth. The finding established PTBP2 as an independent and favorable prognostic factor for NB.

Tumor cells in the deeper regions are deprived of oxygen, nutrients, and growth factors. Therefore, cells grown in 2D cultures under serum starvation can serve as a good model system to study the behavior of tumor cells at the molecular level [155]. In the study by Chae et al., the dose-dependent effects of topotecan on human NB cells under various nutrient supply conditions were investigated, and serum-starved SK-N-SH cells showed unique resistance to topotecan [156]. They performed TMT proteomic and phosphoproteomic analysis on the model system to identify topotecan resistance factors. Functional enrichment and network analysis illustrated the increased DNA repair activity and cholesterol-mediated drug efflux, as well as the activated insulin/mTOR signaling may contribute to the resistance. The upregulation of the three representative proteins BLM, HCR24, and phosphorylated IRS-1 were further confirmed by immunoblotting. This study demonstrated the specific mechanism associated with topotecan resistance and provided a novel model for further investigation in drug resistance.

Cisplatin (CDDP) and/or carboplatin are the most common agents used in NB therapy but also plagued with chemoresistance [157]. Merlos Rodrigo et al. performed a proteomic comparison between UKF-NB-4 cells and their cisplatin-resistant counterpart, UKF-NB-4CDDP cells [158]. Among the significantly different proteomic signatures, functional analyses revealed that UKF-NB-4CDDP cells exhibited a marked increase in proteasome activity and demonstrated significant upregulation of various proteasomal complex subunits, including PSME2, PSMB2, PSMD7, PSMB1, PSMD12, PSMB7, PSMA3 and PSMB5. UKF-NB-4CDDP cells also exhibited up-regulation of proteins involved in various aspects of extracellular transport. These findings support further investigation into combination therapy with CDDP by targeting the lysosomal/proteasomal pathways to improve treatment efficacy in chemoresistant high-risk NB.

**Table 4:** NB study on drug resistance identified by MS-proteomics and major discoveries in past five years.

Authors	Samples	MS Proteomic Methods	Drugs	Protein Targets and the Response to Chemoresistant Status	Related Mechanisms Associated with Drug Resistance
Wang et al. [152]	SK-N-BE(1) and SK-N-BE(2) cells	SILAC-labeled MS	-	ANXA2 (+)	Inducing NF-κB signaling and drug resistance
Tang et al. [154]	Tumors from NB patients	TMT-labled MS	-	PTBP2 (−)	Inducing alternative splicing pathway and repolarization of monocytes
Chae et al. [156]	SK-N-SH cells	TMT-labeled proteomics, phosphoproteomics	Topotecan	BLM (+), HCR24 (+), and phospho-IRS1 (+)	Activating DNA repair, cholesterol-mediated activity, and insulin/mTOR signaling
Merlos Rodrigo et al. [158]	UKF-NB-4 cells	Label-free proteomics	Cisplatin	Proteasomal complex subunits (+)	Activating lysosomal/proteasomal pathways

(+): Up-regulation; (−): Down-regulation; ANXA2: annexin A2; PTBP2: polypyrimidine tract binding protein 2.

## 4. Proteomics Application in Intratumor Heterogeneity of NB

### 4.1. Genetic and Proteomic Level Analysis in NB Intratumor Heterogeneity

Intratumor heterogeneity (ITH) refers to the existence of diverse subpopulations of cells within a single tumor, displaying variations at the genetic, epigenetic, and proteomic levels [159,160]. Multiple cellular subpopulations are easy to be found in high-risk NB, with genetic and epigenetic differences [161]. In early studies, the NB tumor cell phenotypes were described into three types: N-type (neuronal), S-type (substrate adherent), and I-type (intermediary) cells, with different cell culture behavior and protein expression pattern, such as membrane-GD2 and nuclear-calcyclin [83,162]. Recent study has regrouped NB into four distinct epigenetic subtypes based on the super-enhancer landscape and *MYCN* amplification status: *MYCN*-amplified high-risk, *MYCN* non-amplified high-risk, *MYCN* non-amplified low-risk, and mesenchymal-type (MES). The fourth type exhibiting mesenchymal characteristics, was induced by RAS activation and enriched in relapsed cases [163]. Significant efforts have been dedicated to characterizing genomic alterations and oncogenic pathways through comprehensive DNA and RNA sequencing analyses involving thousands of cases. There are many molecular signatures well-characterized in NB cellular subtype detection and risk definition, including *MYCN* amplification, mutations in *ALK*, *PHOX2B*, epigenetic factor such as *ATRX*, and *TERT*, and structural chromosomal changes [2,79,164]. Furthermore, the molecular mechanisms underlying trans-differentiation in NB may elucidate the observed plasticity and intratumoral heterogeneity, including activation of the NOTCH signaling pathway, RAS signaling, and inactivation of *ARID1A* [165,166,167].

MS-based profiling has revealed extensive proteomic heterogeneity within NB tumors. In a study on genetic ITH in NB, ten orthotopic xenograft (PDOX) models from a single primary tumor were subjected to comprehensive proteomic and phosphoproteomic analyses, revealing the spatial expression patterns and diverse molecular profiles involved in stromal contribution, neuronal differentiation, and axon guidance [168]. However, the limited availability of tissues hampers PDX models developing as an important tool for ITH assessment in NB. Furthermore, the differential protein expression patterns among various NB subclones, highlighting the functional diversity within the tumor microenvironment. For instance, recent studies have shown that exosomes could be one way by different clonal populations to interact with each other and shape cellular plasticity [62], and proteomics analysis compared the protein profiling of exosome may contribute to understanding of proteomic diversity in ITH. Moreover, MS-based proteomic approaches can be utilized to investigate distinct PTM patterns, including phosphorylation and ubiquitination, as well as protein stability and interactions, all of which contribute to the functional diversity of NB subclones

### 4.2. The Application of Emerging Proteomic Techniques in NB Intratumor Heterogeneity

The emerging instrumental and bioinformatic tools with cell-type or spatial resolving power have facilitated the exploration of spatial proteome profiles relevant to tumor heterogeneity. Compared to traditional bulk proteomics, which provides an average assessment of protein expression profile, the single cell proteomics (SCP) achieves cell specific proteome data revealing functional diversity in tissue microenvironment [169]. The primary challenge in SCP lies in separating the single cell from the tissue samples with low protein loss [170]. The current methodologies employed for obtaining single cells or cell groups prior to SCP analysis, include fluorescence-activated cell sorting (FACS), magnetic-activated cell sorting (MACS), laser capture microdissection (LCM), capillary electrophoresis and manual cell picking/micromanipulation [171]. Nevertheless, the utilization of SCP technology in NB research is presently restricted, thus requiring the further development.

On the other hand, proteomic patterns can be portrayed directly on tissue slides without isolating cells, therefore preserving critical spatial traits of the microenvironment. This is achieved by mass spectrometry imaging (MSI) using in situ ionization techniques, such as matrix-assisted laser desorption ionization (MALDI), to delineate the spatial distribution of analytes, including drugs, metabolites and peptides [172]. Ryu et al. used MALDI-MSI to analyze the distribution of alectinib (an ALK inhibitor) in NB1 (with ALK amplification) and SK-N-FI (ALK wild-type) NB xenograft tissues [173]. The intra-tumoral abundance of alectinib was quantified using ion signal intensities from MALDI-MSI and normalized by LC-MS/MS. Their findings demonstrated that the distribution pattern of alectinib within xenografted tumors is characterized by heterogeneity, indicating that the distribution of drugs in patient tumors is more complex than previously thought. Therefore, MSI can serve as a valuable tool for assessing drug exposure during early clinical trials and clinical practice, while also identifying regions with low penetration levels that may contribute to drug failure or resistance. In another study, Wu et al. performed MALDI-MSI to inquire spatial heterogeneity of peptides in NB tissues as factors for high and low/intermediate risk classification [174]. Peptides from AHNAK nucleoprotein and collapsin response mediator protein 1 (CRMP1) were identified, which were associated with divergent risks and were further validated immunohistochemically. This study highlights the capability of MALDI-MSI combined with univariate and multivariate analysis strategies to identify spatially risk-associated peptide signatures in NB tissues, thereby providing new biological insights into NB ITH.

## 5. Conclusions and Future Perspective

**Figure 2:**
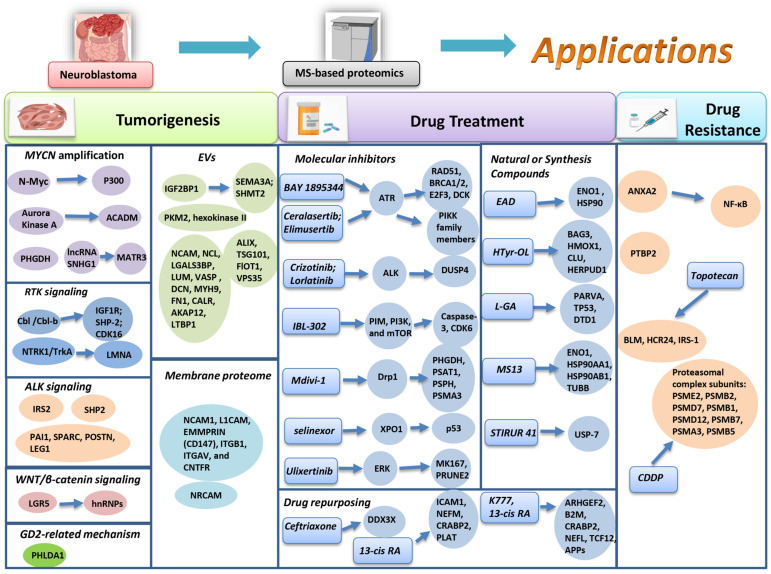
The diagram on the MS-based proteomics application in key protein targets identification in recent five years for NB research. The columns “Tumorigenesis”, “drug treatment”, “drug resistance” are corresponding to protein/gene targets and related drugs listed in Table 1, Table 2 and Table 3, respectively. Protein targets were indicated in circle; drug names were indicated in square; the arrows indicated the potential relationship between the components. ACADM: medium-chain specific acyl-CoA dehydrogenase; PHGDH: phosphoglycerate dehydrogenase; IGF1R: Insulin-like growth factor 1 receptor; SHP2: SH2 domain-containing protein tyrosine phosphatase-2; Cbl: casitas B-lineage lymphoma proteins; IRS2: insulin receptor substrate 2; PAI1: plasminogen activator inhibitor 1; SPARC: secreted protein acidic and cysteine rich; POSTN: periostin; LEG1: galectin-1; LGR5: leucine-rich repeat-containing G-protein coupled receptor 5; IGF2BP1: Insulin-like growth factor 2 mRNA-binding protein 1; SEMA3A: semaphorin 3A; SHMT2: mitochondrial serine hydroxymethyl transferase 2; PKM2: pyruvate kinase M2; HK2: hexokinase II; NCAM: neural cell adhesion molecule; NCL: nucleolin; LGALS3BP: galectin-3-binding protein; LUM: lumican; VASP: vasodilator stimulated phosphoprotein; DCN: decorin; MYH9: myosin-9; FN1: fibronectin; LTBP1: latent-transforming growth factor-beta-binding protein 1; CALR: calreticulin; AKAP12: A-kinase anchor protein 12; NRCAM: neuroglia related cell adhesion molecule; ATR: ataxia telangiectasia and Rad3-related protein; PIKK: phosphoinositide three-kinase-related kinase; ALK: anaplastic lymphoma kinase; Mdivi-1: mitochondrial division inhibitor 1; Drp1: dynamin-related protein 1; XPO1: exportin-1; EAD: epoxyazadiradione; HTyr-OL: hydroxytyrosyl oleate; L-GA: L-glyceraldehyde; ENO1: enolase1; TUBB: tubulin beta chain; USP-7: ubiquitin carboxyl-terminal hydrolase 7; 13-cis RA: 13-cis retinoic acid; ANXA2: annexin A2; PTBP2: polypyrimidine tract binding protein 2.

NB is the most common extracranial cancer affecting children. To improve clinical outcomes for children with high-risk NB, the MS-based proteomic approach, with the dramatic advance of application in recent years, has become a mainstream approach for exploring molecular mechanisms in NB. This article reviews the application of proteomics in NB over the past five years, focusing on diverse aspects such as tumorigenesis, drug treatment and resistance in NB and highlighting many promising protein signatures and molecular mechanisms that may promote clinical research in NB (Figure 2). Furthermore, based on the conclusions drawn from these referenced articles, Table 5 provides a detailed representation of the main protein signatures associated with potential molecular risk factors and risk-related mechanisms of NB. *MYCN* network and status, RTK/ALK signaling pathway, PI3K/Akt/mTOR pathway, WNT/β-catenin signaling, RAS/MAPK signaling pathway, and ATR signaling are highlighted as key risk factors in NB in our review.

Many of these protein findings are considered indispensable in NB tumorigenesis and prognosis, as they have been identified by multiple researchers and implicated in intersecting signaling pathways or drug mechanisms. For instance, SHP2 is an oncogenic tyrosine phosphatase encoded by PTPN11, exhibiting a significant mutation frequency (3.4% of NB cases) and extensively studied as a potential target for inhibiting NB tumor cell growth [175]. Hsp90 is indispensable for the stability and function of many client proteins, emerging as an important target in a variety of cancers [176], including NB growth [177]. PHGDH is an essential enzyme for de novo serine synthesis and has been found to correlate with poor prognosis and potential therapeutic option in many cancers, including MYCN-amplified NB [178]. NCAM is a neural cell adhesion molecule linked to metastatic capacity and aggressive cancer progression in NB. Not only NCAM but also other cell adhesion molecules (CAMs) play pivotal roles in multiple biological processes, necessitating research on CAMs to explore novel targeted therapies in NB. On the other hand, certain distinct protein findings identified across studies may be involved in common biological processes and signaling pathways. For example, IGF1R, IRS2, SHP2 and NTRK1 are prominent molecular signatures in RTK/ALK signaling associated with high-risk NB; PIKK family [179], PRUNE2 [128], E2F transcription factors [180], RAD51, BRCA1/2, BLM and HCR24 [156] are key components associated with DNA damage/repair machinery and cell cycle progression; PKM2, hexokinase II [61] and ENO1 [181] are glycolytic enzymes contributing to glycolysis metabolism; ICAM1, NEFM, CRABP2, PLAT are recognized as important components in neurofilament formation and neuronal differentiation [136,137].

Notably, MS-based proteomics methods are commonly customized for different sample types (cell lines, tissue from human or mouse model, biological fluids), and perturbations (gene overexpression or depletion, inhibitor or activator treatments). For some specific proteomics applications, special optimizations are needed. For instance, the co-IP and RNA pull-down assay combined with LC-MS/MS were conventional methods for investigating protein/RNA interacting proteins [49,52]. Integrated proximal proteomics (IPP) strategy [55] and proximity-dependent biotin identification (BioID)-Screening method [56] are novel proteomics strategies mentioned for interactome investigation. What is more, the protein extraction and purification methods in pre-treatment are critical for proteomics study, particular in PTMs identification, EVs or membrane proteome investigation.

Nonetheless, there are significant obstacles that need to be addressed for proteomics techniques to transition from limited experimental use to broader into more practical clinical applications in NB. The tumor heterogeneity poses a significant challenge. SCP and spatial proteomics are new approaches that deserve further development in the future. Moreover, the identification of low-abundance proteins remains a hurdle in deep proteomic investigations. Current techniques such as centrifugal ultrafiltration, organic solvent precipitation, electrophoresis, and chromatography can be employed to enhance the detection of low-abundance proteins, and more development in this field should be expected [182]. Furthermore, while proteomics has facilitated the discovery and evaluation of potential disease biomarkers using large-scale clinical sample cohorts in many other diseases, its application in NB diagnosis and prognosis research is still relatively nascent, potentially hampered by the limited availability of clinical NB samples. Encouragingly, advancements of new hardware with higher sensitivity, and novel sample preparation workflows designed for minimal sample requirement, are anticipated to drive more proteomics-driven studies in this area and eventually lead to exciting breakthroughs for NB treatment in the future.

## Figures and Tables

**Table 5:** a. Major protein findings related to molecular risk factors in NB. b. Major protein findings related to risk stratification in NB.

**a. Molecular risk factors in NB**	**Relevant protein targets in this review**
*MYCN* network or *MYCN* status	P300 [49], PHGDH [51], PKM2 [61], HK2 [61], Alix [62], TSG101 [62], FLOT1 [62], VPS35 [62], DDX3X [135],
RTK/ALK signaling	IGF1R [53], SHP2 [53,56], CDK16 [53], LMNA [54], IRS2 [55], DUSP4 [105]
Wnt/β-catenin signaling	hnRNPH3, hnRNPA2B1 [58]
RAS/MAPK pathway	SHP2 [53,56], PRUNE2 [127], MK167 [127], DUSP4 [105]
ATR activity	RAD51 [101], BRCA1/2 [101], E2F3 [101], DCK [101], ATM [102], DNAPK [102]
PI3K/Akt/mTOR pathway	IRS2 [55], Caspase3 [110], CDK6 [110], PIKK Family [102]
**b. Risk stratification in NB**	**Relevant protein targets in this review**
High risk-related	P300 [49], PHGDH [51,113], MATR3 [52], SHP2 [53,56], PAI1 [57], SPARC [57], POSTN [57], LEG1 [57], IRS2 [55], SEMA3A [60], SHMT2 [60], PKM2 [61], HK2 [61], NCAM [63,64], NCL [63], LGALS3BP [63], MYH9 [63], FN1 [63], LTBP1 [63], DDX3X [135], ANXA2 [152]
Favorable outcome-related	ACADM [50], LUM [63], VASP [63], DCN [63], CALR [63], AKAP12 [63], PRUNE2 [127], Caspase3 [110], PTBP2 [154]

PHGDH: phosphoglycerate dehydrogenase; PKM2: pyruvate kinase M2; HK2: hexokinase II; IGF1R: Insulin-like growth factor 1 receptor; SHP2: SH2 domain-containing protein tyrosine phosphatase-2; IRS2: insulin receptor substrate 2; ATM: ataxia-telangiectasia mutated; DNAPK: DNA-dependent protein kinase; PIKK: phosphoinositide three-kinase-related kinase; PAI1: plasminogen activator inhibitor 1; SPARC: secreted protein acidic and cysteine rich; POSTN: periostin; LEG1: galectin-1; SEMA3A: semaphorin 3A; SHMT2: mitochondrial serine hydroxymethyl transferase 2; NCAM: neural cell adhesion molecule; NCL: nucleolin; LGALS3BP: galectin-3-binding protein; MYH9: myosin-9; FN1: fibronectin; LTBP1: latent-transforming growth factor-beta-binding protein 1; ANXA2: annexin A2; CALR: calreticulin; AKAP12: A-kinase anchor protein 12; ACADM: medium-chain specific acyl-CoA dehydrogenase; LUM: lumican; VASP: vasodilator stimulated phosphoprotein; DCN: decorin; PTBP2: polypyrimidine tract binding protein 2.

## Data Availability

Not applicable.

## References

[1] Maris J.M., Hogarty M.D., Bagatell R., Cohn S.L. (2007). Neuroblastoma. Lancet.

[2] Zafar A., Wang W., Liu G., Wang X., Xian W., McKeon F., Foster J., Zhou J., Zhang R. (2021). Molecular targeting therapies for neuroblastoma: Progress and challenges. Med. Res. Rev..

[3] Mlakar V., Jurkovic Mlakar S., Lopez G., Maris J.M., Ansari M., Gumy-Pause F. (2017). 11q deletion in neuroblastoma: A review of biological and clinical implications. Mol. Cancer.

[4] Maris J.M. (2010). Recent advances in neuroblastoma. N. Engl. J. Med..

[5] Whittle S.B., Smith V., Doherty E., Zhao S., McCarty S., Zage P.E. (2017). Overview and recent advances in the treatment of neuroblastoma. Expert Rev. Anticancer Ther..

[6] Baker D.L., Schmidt M.L., Cohn S.L., Maris J.M., London W.B., Buxton A., Stram D., Castleberry R.P., Shimada H., Sandler A. (2010). Outcome after reduced chemotherapy for intermediate-risk neuroblastoma. N. Engl. J. Med..

[7] Kholodenko I.V., Kalinovsky D.V., Doronin I.I., Deyev S.M., Kholodenko R.V. (2018). Neuroblastoma Origin and Therapeutic Targets for Immunotherapy. J. Immunol. Res..

[8] Swift C.C., Eklund M.J., Kraveka J.M., Alazraki A.L. (2018). Updates in Diagnosis, Management, and Treatment of Neuroblastoma. Radiographics.

[9] Qiu B., Matthay K.K. (2022). Advancing therapy for neuroblastoma. Nat. Rev. Clin. Oncol..

[10] Schleiermacher G., Janoueix-Lerosey I., Delattre O. (2014). Recent insights into the biology of neuroblastoma. Int. J. Cancer.

[11] Shawraba F., Hammoud H., Mrad Y., Saker Z., Fares Y., Harati H., Bahmad H.F., Nabha S. (2021). Biomarkers in Neuroblastoma: An Insight into Their Potential Diagnostic and Prognostic Utilities. Curr. Treat. Options Oncol..

[12] Monclair T., Brodeur G.M., Ambros P.F., Brisse H.J., Cecchetto G., Holmes K., Kaneko M., London W.B., Matthay K.K., Nuchtern J.G. (2009). The International Neuroblastoma Risk Group (INRG) staging system: An INRG Task Force report. J. Clin. Oncol..

[13] Seeger R.C., Brodeur G.M., Sather H., Dalton A., Siegel S.E., Wong K.Y., Hammond D. (1985). Association of multiple copies of the N-myc oncogene with rapid progression of neuroblastomas. N. Engl. J. Med..

[14] Park J.A., Cheung N.V. (2020). Targets and Antibody Formats for Immunotherapy of Neuroblastoma. J. Clin. Oncol..

[15] Cheung C.H.Y., Juan H.F. (2017). Quantitative proteomics in lung cancer. J. Biomed. Sci..

[16] Pandey A., Mann M. (2000). Proteomics to study genes and genomes. Nature.

[17] Smiles W.J., Catalano L., Stefan V.E., Weber D.D., Kofler B. (2023). Metabolic protein kinase signalling in neuroblastoma. Mol. Metab..

[18] Kumar P., Koach J., Nekritz E., Mukherjee S., Braun B.S., DuBois S.G., Nasholm N., Haas-Kogan D., Matthay K.K., Weiss W.A. (2024). Aurora Kinase A inhibition enhances DNA damage and tumor cell death with (131)I-MIBG therapy in high-risk neuroblastoma. EJNMMI Res..

[19] Paccosi E., Costantino M., Balzerano A., Filippi S., Brancorsini S., Proietti-De-Santis L. (2021). Neuroblastoma Cells Depend on CSB for Faithful Execution of Cytokinesis and Survival. Int. J. Mol. Sci..

[20] Wang Z., Gerstein M., Snyder M. (2009). RNA-Seq: A revolutionary tool for transcriptomics. Nat. Rev. Genet..

[21] Martinez-Rodriguez F., Limones-Gonzalez J.E., Mendoza-Almanza B., Esparza-Ibarra E.L., Gallegos-Flores P.I., Ayala-Lujan J.L., Godina-Gonzalez S., Salinas E., Mendoza-Almanza G. (2021). Understanding Cervical Cancer through Proteomics. Cells.

[22] Pascovici D., Wu J.X., McKay M.J., Joseph C., Noor Z., Kamath K., Wu Y., Ranganathan S., Gupta V., Mirzaei M. (2018). Clinically Relevant Post-Translational Modification Analyses-Maturing Workflows and Bioinformatics Tools. Int. J. Mol. Sci..

[23] Conrad D.H., Goyette J., Thomas P.S. (2008). Proteomics as a method for early detection of cancer: A review of proteomics, exhaled breath condensate, and lung cancer screening. J. Gen. Intern. Med..

[24] Alessandro R., Fontana S., Kohn E., De Leo G. (2005). Proteomic strategies and their application in cancer research. Tumori.

[25] Zhang Z., Wu S., Stenoien D.L., Pasa-Tolic L. (2014). High-throughput proteomics. Annu. Rev. Anal. Chem..

[26] Duong V.A., Lee H. (2023). Bottom-Up Proteomics: Advancements in Sample Preparation. Int. J. Mol. Sci..

[27] Miller R.M., Smith L.M. (2023). Overview and considerations in bottom-up proteomics. Analyst.

[28] Ryu J., Thomas S.N. (2021). Quantitative Mass Spectrometry-Based Proteomics for Biomarker Development in Ovarian Cancer. Molecules.

[29] Li K.W., Gonzalez-Lozano M.A., Koopmans F., Smit A.B. (2020). Recent Developments in Data Independent Acquisition (DIA) Mass Spectrometry: Application of Quantitative Analysis of the Brain Proteome. Front. Mol. Neurosci..

[30] Elias J.E., Gygi S.P. (2007). Target-decoy search strategy for increased confidence in large-scale protein identifications by mass spectrometry. Nat. Methods.

[31] Gillet L.C., Navarro P., Tate S., Rost H., Selevsek N., Reiter L., Bonner R., Aebersold R. (2012). Targeted data extraction of the MS/MS spectra generated by data-independent acquisition: A new concept for consistent and accurate proteome analysis. Mol. Cell Proteom..

[32] Yang J., Liu Q., Yu B., Han B., Yang B. (2022). 4D-quantitative proteomics signature of asthenozoospermia and identification of extracellular matrix protein 1 as a novel biomarker for sperm motility. Mol. Omics.

[33] Pei Y., Chen S., Zhang Y., Olga V., Li Y., Diao X., Zhou H. (2022). Coral and it’s symbionts responses to the typical global marine pollutant BaP by 4D-Proteomics approach. Environ. Pollut..

[34] Meier F., Brunner A.D., Frank M., Ha A., Bludau I., Voytik E., Kaspar-Schoenefeld S., Lubeck M., Raether O., Bache N. (2020). diaPASEF: Parallel accumulation-serial fragmentation combined with data-independent acquisition. Nat. Methods.

[35] Zhang B., VerBerkmoes N.C., Langston M.A., Uberbacher E., Hettich R.L., Samatova N.F. (2006). Detecting differential and correlated protein expression in label-free shotgun proteomics. J. Proteome Res..

[36] Ong S.E., Blagoev B., Kratchmarova I., Kristensen D.B., Steen H., Pandey A., Mann M. (2002). Stable isotope labeling by amino acids in cell culture, SILAC, as a simple and accurate approach to expression proteomics. Mol. Cell Proteom..

[37] Swiatly A., Horala A., Matysiak J., Hajduk J., Nowak-Markwitz E., Kokot Z.J. (2018). Understanding Ovarian Cancer: iTRAQ-Based Proteomics for Biomarker Discovery. Int. J. Mol. Sci..

[38] Zhang L., Elias J.E. (2017). Relative Protein Quantification Using Tandem Mass Tag Mass Spectrometry. Methods Mol. Biol..

[39] Srinivasan A., Sing J.C., Gingras A.C., Rost H.L. (2022). Improving Phosphoproteomics Profiling Using Data-Independent Mass Spectrometry. J. Proteome Res..

[40] Sahu I., Zhu H., Buhrlage S.J., Marto J.A. (2023). Proteomic approaches to study ubiquitinomics. Biochim. Biophys. Acta Gene Regul. Mech..

[41] Peterson A.C., Russell J.D., Bailey D.J., Westphall M.S., Coon J.J. (2012). Parallel reaction monitoring for high resolution and high mass accuracy quantitative, targeted proteomics. Mol. Cell Proteom..

[42] Mann M., Kumar C., Zeng W.F., Strauss M.T. (2021). Artificial intelligence for proteomics and biomarker discovery. Cell Syst..

[43] Zhou X.X., Zeng W.F., Chi H., Luo C., Liu C., Zhan J., He S.M., Zhang Z. (2017). pDeep: Predicting MS/MS Spectra of Peptides with Deep Learning. Anal. Chem..

[44] Geyer P.E., Voytik E., Treit P.V., Doll S., Kleinhempel A., Niu L., Muller J.B., Buchholtz M.L., Bader J.M., Teupser D. (2019). Plasma Proteome Profiling to detect and avoid sample-related biases in biomarker studies. EMBO Mol. Med..

[45] Ge J., Ge J., Tang G., Xiong D., Zhu D., Ding X., Zhou X., Sang M. (2024). Machine learning-based identification of biomarkers and drugs in immunologically cold and hot pancreatic adenocarcinomas. J. Transl. Med..

[46] Lee T., Natalwala J., Chapple V., Liu Y. (2024). A brief history of artificial intelligence embryo selection: From black-box to glass-box. Hum. Reprod..

[47] Sen P., Lamichhane S., Mathema V.B., McGlinchey A., Dickens A.M., Khoomrung S., Oresic M. (2021). Deep learning meets metabolomics: A methodological perspective. Brief. Bioinform..

[48] Cifani P., Kentsis A. (2017). Towards comprehensive and quantitative proteomics for diagnosis and therapy of human disease. Proteomics.

[49] Cheng C., He T., Chen K., Cai Y., Gu Y., Pan L., Duan P., Wu Y., Wu Z. (2023). P300 Interacted with N-Myc and Regulated Its Protein Stability via Altering Its Post-Translational Modifications in Neuroblastoma. Mol. Cell Proteom..

[50] Hsieh C.H., Cheung C.H.Y., Liu Y.L., Hou C.L., Hsu C.L., Huang C.T., Yang T.S., Chen S.F., Chen C.N., Hsu W.M. (2019). Quantitative Proteomics of Th-MYCN Transgenic Mice Reveals Aurora Kinase Inhibitor Altered Metabolic Pathways and Enhanced ACADM To Suppress Neuroblastoma Progression. J. Proteome Res..

[51] Arlt B., Zasada C., Baum K., Wuenschel J., Mastrobuoni G., Lodrini M., Astrahantseff K., Winkler A., Schulte J.H., Finkler S. (2021). Inhibiting phosphoglycerate dehydrogenase counteracts chemotherapeutic efficacy against MYCN-amplified neuroblastoma. Int. J. Cancer.

[52] Yang T.W., Sahu D., Chang Y.W., Hsu C.L., Hsieh C.H., Huang H.C., Juan H.F. (2019). RNA-Binding Proteomics Reveals MATR3 Interacting with lncRNA SNHG1 To Enhance Neuroblastoma Progression. J. Proteome Res..

[53] Pedersen A.K., Pfeiffer A., Karemore G., Akimov V., Bekker-Jensen D.B., Blagoev B., Francavilla C., Olsen J.V. (2021). Proteomic investigation of Cbl and Cbl-b in neuroblastoma cell differentiation highlights roles for SHP-2 and CDK16. iScience.

[54] Funke L., Bracht T., Oeck S., Schork K., Stepath M., Dreesmann S., Eisenacher M., Sitek B., Schramm A. (2021). NTRK1/TrkA Signaling in Neuroblastoma Cells Induces Nuclear Reorganization and Intra-Nuclear Aggregation of Lamin A/C. Cancers.

[55] Emdal K.B., Pedersen A.K., Bekker-Jensen D.B., Lundby A., Claeys S., De Preter K., Speleman F., Francavilla C., Olsen J.V. (2018). Integrated proximal proteomics reveals IRS2 as a determinant of cell survival in ALK-driven neuroblastoma. Sci. Signal.

[56] Uckun E., Siaw J.T., Guan J., Anthonydhason V., Fuchs J., Wolfstetter G., Hallberg B., Palmer R.H. (2021). BioID-Screening Identifies PEAK1 and SHP2 as Components of the ALK Proximitome in Neuroblastoma Cells. J. Mol. Biol..

[57] Li J., Wang Y., Li L., Or P.M., Wai Wong C., Liu T., Ho W.L.H., Chan A.M. (2021). Tumour-derived substrate-adherent cells promote neuroblastoma survival through secreted trophic factors. Mol. Oncol..

[58] Hwang M., Han M.H., Park H.H., Choi H., Lee K.Y., Lee Y.J., Kim J.M., Cheong J.H., Ryu J.I., Min K.W. (2019). LGR5 and Downstream Intracellular Signaling Proteins Play Critical Roles in the Cell Proliferation of Neuroblastoma, Meningioma and Pituitary Adenoma. Exp. Neurobiol..

[59] Bugara B., Durbas M., Kudrycka M., Malinowska A., Horwacik I., Rokita H. (2024). Silencing of the PHLDA1 leads to global proteome changes and differentiation pathways of human neuroblastoma cells. Front. Pharmacol..

[60] Dhamdhere M.R., Gowda C.P., Singh V., Liu Z., Carruthers N., Grant C.N., Sharma A., Dovat S., Sundstrom J.M., Wang H.G. (2023). IGF2BP1 regulates the cargo of extracellular vesicles and promotes neuroblastoma metastasis. Oncogene.

[61] Tsakaneli A., Carregari V.C., Morini M., Eva A., Cangemi G., Chayka O., Makarov E., Bibbo S., Capone E., Sala G. (2021). MYC regulates metabolism through vesicular transfer of glycolytic kinases. Open Biol..

[62] Fonseka P., Liem M., Ozcitti C., Adda C.G., Ang C.S., Mathivanan S. (2019). Exosomes from N-Myc amplified neuroblastoma cells induce migration and confer chemoresistance to non-N-Myc amplified cells: Implications of intra-tumour heterogeneity. J. Extracell. Vesicles.

[63] Morini M., Raggi F., Bartolucci M., Petretto A., Ardito M., Rossi C., Segalerba D., Garaventa A., Eva A., Cangelosi D. (2023). Plasma-Derived Exosome Proteins as Novel Diagnostic and Prognostic Biomarkers in Neuroblastoma Patients. Cells.

[64] Garcia J., Faca V., Jarzembowski J., Zhang Q., Park J., Hanash S. (2009). Comprehensive profiling of the cell surface proteome of Sy5Y neuroblastoma cells yields a subset of proteins associated with tumor differentiation. J. Proteome Res..

[65] Gangras P., Gelfanova V., Williams G.D., Handelman S.K., Smith R.M., Debets M.F. (2022). Investigating SH-SY5Y Neuroblastoma Cell Surfaceome as a Model for Neuronal-Targeted Novel Therapeutic Modalities. Int. J. Mol. Sci..

[66] Misawa A., Hosoi H., Arimoto A., Shikata T., Akioka S., Matsumura T., Houghton P.J., Sawada T. (2000). N-Myc induction stimulated by insulin-like growth factor I through mitogen-activated protein kinase signaling pathway in human neuroblastoma cells. Cancer Res..

[67] Marshall G.M., Liu P.Y., Gherardi S., Scarlett C.J., Bedalov A., Xu N., Iraci N., Valli E., Ling D., Thomas W. (2011). SIRT1 promotes N-Myc oncogenesis through a positive feedback loop involving the effects of MKP3 and ERK on N-Myc protein stability. PLoS Genet..

[68] Otto T., Horn S., Brockmann M., Eilers U., Schuttrumpf L., Popov N., Kenney A.M., Schulte J.H., Beijersbergen R., Christiansen H. (2009). Stabilization of N-Myc is a critical function of Aurora A in human neuroblastoma. Cancer Cell.

[69] Houten S.M., Wanders R.J. (2010). A general introduction to the biochemistry of mitochondrial fatty acid beta-oxidation. J. Inherit. Metab. Dis..

[70] Yang M., Vousden K.H. (2016). Serine and one-carbon metabolism in cancer. Nat. Rev. Cancer.

[71] Sahu D., Hsu C.L., Lin C.C., Yang T.W., Hsu W.M., Ho S.Y., Juan H.F., Huang H.C. (2016). Co-expression analysis identifies long noncoding RNA SNHG1 as a novel predictor for event-free survival in neuroblastoma. Oncotarget.

[72] Edsjo A., Holmquist L., Pahlman S. (2007). Neuroblastoma as an experimental model for neuronal differentiation and hypoxia-induced tumor cell dedifferentiation. Semin. Cancer Biol..

[73] Rozen E.J., Shohet J.M. (2022). Systematic review of the receptor tyrosine kinase superfamily in neuroblastoma pathophysiology. Cancer Metastasis Rev..

[74] Emdal K.B., Pedersen A.K., Bekker-Jensen D.B., Tsafou K.P., Horn H., Lindner S., Schulte J.H., Eggert A., Jensen L.J., Francavilla C. (2015). Temporal proteomics of NGF-TrkA signaling identifies an inhibitory role for the E3 ligase Cbl-b in neuroblastoma cell differentiation. Sci. Signal.

[75] Chen B., Hammonds-Odie L., Perron J., Masters B.A., Bixby J.L. (2002). SHP-2 mediates target-regulated axonal termination and NGF-dependent neurite growth in sympathetic neurons. Dev. Biol..

[76] Dohmen M., Krieg S., Agalaridis G., Zhu X., Shehata S.N., Pfeiffenberger E., Amelang J., Butepage M., Buerova E., Pfaff C.M. (2020). AMPK-dependent activation of the Cyclin Y/CDK16 complex controls autophagy. Nat. Commun..

[77] Pajtler K.W., Mahlow E., Odersky A., Lindner S., Stephan H., Bendix I., Eggert A., Schramm A., Schulte J.H. (2014). Neuroblastoma in dialog with its stroma: NTRK1 is a regulator of cellular cross-talk with Schwann cells. Oncotarget.

[78] Pacenta H.L., Macy M.E. (2018). Entrectinib and other ALK/TRK inhibitors for the treatment of neuroblastoma. Drug Des. Dev. Ther..

[79] Pugh T.J., Morozova O., Attiyeh E.F., Asgharzadeh S., Wei J.S., Auclair D., Carter S.L., Cibulskis K., Hanna M., Kiezun A. (2013). The genetic landscape of high-risk neuroblastoma. Nat. Genet..

[80] Mosse Y.P., Laudenslager M., Longo L., Cole K.A., Wood A., Attiyeh E.F., Laquaglia M.J., Sennett R., Lynch J.E., Perri P. (2008). Identification of ALK as a major familial neuroblastoma predisposition gene. Nature.

[81] Guan J., Tucker E.R., Wan H., Chand D., Danielson L.S., Ruuth K., El Wakil A., Witek B., Jamin Y., Umapathy G. (2016). The ALK inhibitor PF-06463922 is effective as a single agent in neuroblastoma driven by expression of ALK and MYCN. Dis. Model. Mech..

[82] Shaw L.M. (2011). The insulin receptor substrate (IRS) proteins: At the intersection of metabolism and cancer. Cell Cycle.

[83] Ross R.A., Biedler J.L., Spengler B.A. (2003). A role for distinct cell types in determining malignancy in human neuroblastoma cell lines and tumors. Cancer Lett..

[84] Vieira G.C., Chockalingam S., Melegh Z., Greenhough A., Malik S., Szemes M., Park J.H., Kaidi A., Zhou L., Catchpoole D. (2015). LGR5 regulates pro-survival MEK/ERK and proliferative Wnt/beta-catenin signalling in neuroblastoma. Oncotarget.

[85] Larrosa C., Mora J., Cheung N.K. (2023). Global Impact of Monoclonal Antibodies (mAbs) in Children: A Focus on Anti-GD2. Cancers.

[86] Ladenstein R., Potschger U., Valteau-Couanet D., Luksch R., Castel V., Yaniv I., Laureys G., Brock P., Michon J.M., Owens C. (2018). Interleukin 2 with anti-GD2 antibody ch14.18/CHO (dinutuximab beta) in patients with high-risk neuroblastoma (HR-NBL1/SIOPEN): A multicentre, randomised, phase 3 trial. Lancet Oncol..

[87] Philippova J., Shevchenko J., Sennikov S. (2024). GD2-targeting therapy: A comparative analysis of approaches and promising directions. Front. Immunol..

[88] Kaczanowska S., Murty T., Alimadadi A., Contreras C.F., Duault C., Subrahmanyam P.B., Reynolds W., Gutierrez N.A., Baskar R., Wu C.J. (2024). Immune determinants of CAR-T cell expansion in solid tumor patients receiving GD2 CAR-T cell therapy. Cancer Cell.

[89] Horwacik I., Durbas M., Boratyn E., Sawicka A., Wegrzyn P., Krzanik S., Gorka A., Drozniak J., Augustyniak E., Kowalczyk A. (2015). Analysis of genes involved in response to doxorubicin and a GD2 ganglioside-specific 14G2a monoclonal antibody in IMR-32 human neuroblastoma cells. Acta Biochim. Pol..

[90] Dhamdhere M.R., Spiegelman V.S. (2024). Extracellular vesicles in neuroblastoma: Role in progression, resistance to therapy and diagnostics. Front. Immunol..

[91] van Niel G., Carter D.R.F., Clayton A., Lambert D.W., Raposo G., Vader P. (2022). Challenges and directions in studying cell-cell communication by extracellular vesicles. Nat. Rev. Mol. Cell Biol..

[92] Azmi A.S., Bao B., Sarkar F.H. (2013). Exosomes in cancer development, metastasis, and drug resistance: A comprehensive review. Cancer Metastasis Rev..

[93] Guo Y., Ji X., Liu J., Fan D., Zhou Q., Chen C., Wang W., Wang G., Wang H., Yuan W. (2019). Effects of exosomes on pre-metastatic niche formation in tumors. Mol. Cancer.

[94] Biegel J.M., Dhamdhere M., Gao S., Gowda C.P., Kawasawa Y.I., Spiegelman V.S. (2021). Inhibition of the mRNA-Binding Protein IGF2BP1 Suppresses Proliferation and Sensitizes Neuroblastoma Cells to Chemotherapeutic Agents. Front. Oncol..

[95] Casey S.C., Baylot V., Felsher D.W. (2018). The MYC oncogene is a global regulator of the immune response. Blood.

[96] Gangoda L., Boukouris S., Liem M., Kalra H., Mathivanan S. (2015). Extracellular vesicles including exosomes are mediators of signal transduction: Are they protective or pathogenic?. Proteomics.

[97] Li N., Spetz M.R., Li D., Ho M. (2021). Advances in immunotherapeutic targets for childhood cancers: A focus on glypican-2 and B7-H3. Pharmacol. Ther..

[98] Waas M., Snarrenberg S.T., Littrell J., Jones Lipinski R.A., Hansen P.A., Corbett J.A., Gundry R.L. (2020). SurfaceGenie: A web-based application for prioritizing cell-type-specific marker candidates. Bioinformatics.

[99] Saldivar J.C., Cortez D., Cimprich K.A. (2017). Publisher correction: The essential kinase ATR: Ensuring faithful duplication of a challenging genome. Nat. Rev. Mol. Cell Biol..

[100] Gilad O., Nabet B.Y., Ragland R.L., Schoppy D.W., Smith K.D., Durham A.C., Brown E.J. (2010). Combining ATR suppression with oncogenic Ras synergistically increases genomic instability, causing synthetic lethality or tumorigenesis in a dosage-dependent manner. Cancer Res..

[101] Szydzik J., Lind D.E., Arefin B., Kurhe Y., Umapathy G., Siaw J.T., Claeys A., Gabre J.L., Van den Eynden J., Hallberg B. (2021). ATR inhibition enables complete tumour regression in ALK-driven NB mouse models. Nat. Commun..

[102] Borenas M., Umapathy G., Lind D.E., Lai W.Y., Guan J., Johansson J., Jennische E., Schmidt A., Kurhe Y., Gabre J.L. (2024). ALK signaling primes the DNA damage response sensitizing ALK-driven neuroblastoma to therapeutic ATR inhibition. Proc. Natl. Acad. Sci. USA.

[103] Mosse Y.P. (2016). Anaplastic Lymphoma Kinase as a Cancer Target in Pediatric Malignancies. Clin. Cancer Res..

[104] Lin J.J., Riely G.J., Shaw A.T. (2017). Targeting ALK: Precision Medicine Takes on Drug Resistance. Cancer Discov..

[105] Van den Eynden J., Umapathy G., Ashouri A., Cervantes-Madrid D., Szydzik J., Ruuth K., Koster J., Larsson E., Guan J., Palmer R.H. (2018). Phosphoproteome and gene expression profiling of ALK inhibition in neuroblastoma cell lines reveals conserved oncogenic pathways. Sci. Signal.

[106] Chesler L., Schlieve C., Goldenberg D.D., Kenney A., Kim G., McMillan A., Matthay K.K., Rowitch D., Weiss W.A. (2006). Inhibition of phosphatidylinositol 3-kinase destabilizes Mycn protein and blocks malignant progression in neuroblastoma. Cancer Res..

[107] Segerstrom L., Baryawno N., Sveinbjornsson B., Wickstrom M., Elfman L., Kogner P., Johnsen J.I. (2011). Effects of small molecule inhibitors of PI3K/Akt/mTOR signaling on neuroblastoma growth in vitro and in vivo. Int. J. Cancer.

[108] Le X., Antony R., Razavi P., Treacy D.J., Luo F., Ghandi M., Castel P., Scaltriti M., Baselga J., Garraway L.A. (2016). Systematic Functional Characterization of Resistance to PI3K Inhibition in Breast Cancer. Cancer Discov..

[109] Nawijn M.C., Alendar A., Berns A. (2011). For better or for worse: The role of Pim oncogenes in tumorigenesis. Nat. Rev. Cancer.

[110] Mohlin S., Hansson K., Radke K., Martinez S., Blanco-Apiricio C., Garcia-Ruiz C., Welinder C., Esfandyari J., O’Neill M., Pastor J. (2019). Anti-tumor effects of PIM/PI3K/mTOR triple kinase inhibitor IBL-302 in neuroblastoma. EMBO Mol. Med..

[111] Kim H., Lee J.Y., Park K.J., Kim W.H., Roh G.S. (2016). A mitochondrial division inhibitor, Mdivi-1, inhibits mitochondrial fragmentation and attenuates kainic acid-induced hippocampal cell death. BMC Neurosci..

[112] Dai W., Wang G., Chwa J., Oh M.E., Abeywardana T., Yang Y., Wang Q.A., Jiang L. (2020). Mitochondrial division inhibitor (mdivi-1) decreases oxidative metabolism in cancer. Br. J. Cancer.

[113] Wang W.H., Kao Y.C., Hsieh C.H., Tsai S.Y., Cheung C.H.Y., Huang H.C., Juan H.F. (2024). Multiomics Reveals Induction of Neuroblastoma SK-N-BE(2)C Cell Death by Mitochondrial Division Inhibitor 1 through Multiple Effects. J. Proteome Res..

[114] Ryan J., Tivnan A., Fay J., Bryan K., Meehan M., Creevey L., Lynch J., Bray I.M., O’Meara A., Tracey L. (2012). MicroRNA-204 increases sensitivity of neuroblastoma cells to cisplatin and is associated with a favourable clinical outcome. Br. J. Cancer.

[115] Ooi C.Y., Carter D.R., Liu B., Mayoh C., Beckers A., Lalwani A., Nagy Z., De Brouwer S., Decaesteker B., Hung T.T. (2018). Network Modeling of microRNA-mRNA Interactions in Neuroblastoma Tumorigenesis Identifies miR-204 as a Direct Inhibitor of MYCN. Cancer Res..

[116] Bachetti T., Di Zanni E., Ravazzolo R., Ceccherini I. (2015). *miR*-204 mediates post-transcriptional down-regulation of PHOX2B gene expression in neuroblastoma cells. Biochim. Biophys. Acta.

[117] Hong D.S., Kang Y.K., Borad M., Sachdev J., Ejadi S., Lim H.Y., Brenner A.J., Park K., Lee J.L., Kim T.Y. (2020). Phase 1 study of MRX34, a liposomal miR-34a mimic, in patients with advanced solid tumours. Br. J. Cancer.

[118] Usman W.M., Pham T.C., Kwok Y.Y., Vu L.T., Ma V., Peng B., Chan Y.S., Wei L., Chin S.M., Azad A. (2018). Efficient RNA drug delivery using red blood cell extracellular vesicles. Nat. Commun..

[119] Chiangjong W., Panachan J., Keadsanti S., Newburg D.S., Morrow A.L., Hongeng S., Chutipongtanate S. (2024). Development of red blood cell-derived extracellular particles as a biocompatible nanocarrier of microRNA-204 (REP-204) to harness anti-neuroblastoma effect. Nanomedicine.

[120] Yang Y., Guo L., Chen L., Gong B., Jia D., Sun Q. (2023). Nuclear transport proteins: Structure, function, and disease relevance. Signal Transduct. Target. Ther..

[121] Galinski B., Luxemburg M., Landesman Y., Pawel B., Johnson K.J., Master S.R., Freeman K.W., Loeb D.M., Hebert J.M., Weiser D.A. (2021). XPO1 inhibition with selinexor synergizes with proteasome inhibition in neuroblastoma by targeting nuclear export of IkB. Transl. Oncol..

[122] Chari A., Vogl D.T., Gavriatopoulou M., Nooka A.K., Yee A.J., Huff C.A., Moreau P., Dingli D., Cole C., Lonial S. (2019). Oral Selinexor-Dexamethasone for Triple-Class Refractory Multiple Myeloma. N. Engl. J. Med..

[123] Nguyen R., Wang H., Sun M., Lee D.G., Peng J., Thiele C.J. (2022). Combining selinexor with alisertib to target the p53 pathway in neuroblastoma. Neoplasia.

[124] Katayama H., Sasai K., Kawai H., Yuan Z.M., Bondaruk J., Suzuki F., Fujii S., Arlinghaus R.B., Czerniak B.A., Sen S. (2004). Phosphorylation by aurora kinase A induces Mdm2-mediated destabilization and inhibition of p53. Nat. Genet..

[125] Kohno M., Pouyssegur J. (2006). Targeting the ERK signaling pathway in cancer therapy. Ann. Med..

[126] Eleveld T.F., Oldridge D.A., Bernard V., Koster J., Colmet Daage L., Diskin S.J., Schild L., Bentahar N.B., Bellini A., Chicard M. (2015). Relapsed neuroblastomas show frequent RAS-MAPK pathway mutations. Nat. Genet..

[127] Yu Y., Zhao Y., Choi J., Shi Z., Guo L., Elizarraras J., Gu A., Cheng F., Pei Y., Lu D. (2022). ERK Inhibitor Ulixertinib Inhibits High-Risk Neuroblastoma Growth In Vitro and In Vivo. Cancers.

[128] Islam M.S., Takano R., Yokochi T., Akter J., Nakamura Y., Nakagawara A., Tatsumi Y. (2019). Programmed expression of pro-apoptotic BMCC1 during apoptosis, triggered by DNA damage in neuroblastoma cells. BMC Cancer.

[129] Scholzen T., Gerdes J. (2000). The Ki-67 protein: From the known and the unknown. J. Cell Physiol..

[130] Chandel S., Bhattacharya A., Gautam A., Zeng W., Alka O., Sachsenberg T., Gupta G.D., Narang R.K., Ravichandiran V., Singh R. (2023). Investigation of the anti-cancer potential of epoxyazadiradione in neuroblastoma: Experimental assays and molecular analysis. J. Biomol. Struct. Dyn..

[131] Laghezza Masci V., Bernini R., Villanova N., Clemente M., Cicaloni V., Tinti L., Salvini L., Taddei A.R., Tiezzi A., Ovidi E. (2022). In Vitro Anti-Proliferative and Apoptotic Effects of Hydroxytyrosyl Oleate on SH-SY5Y Human Neuroblastoma Cells. Int. J. Mol. Sci..

[132] Forbes M., Kempa R., Mastrobuoni G., Rayman L., Pietzke M., Bayram S., Arlt B., Spruessel A., Deubzer H.E., Kempa S. (2024). L-Glyceraldehyde Inhibits Neuroblastoma Cell Growth via a Multi-Modal Mechanism on Metabolism and Signaling. Cancers.

[133] Lee Y.Q., Rajadurai P., Abas F., Othman I., Naidu R. (2021). Proteomic Analysis on Anti-Proliferative and Apoptosis Effects of Curcumin Analog, 1,5-bis(4-Hydroxy-3-Methyoxyphenyl)-1,4-Pentadiene-3-One-Treated Human Glioblastoma and Neuroblastoma Cells. Front. Mol. Biosci..

[134] Morretta E., Brullo C., Belvedere R., Petrella A., Spallarossa A., Monti M.C. (2023). Targeting USP-7 by a Novel Fluorinated 5-Pyrazolyl-Urea Derivative. Int. J. Mol. Sci..

[135] Chittavanich P., Saengwimol D., Roytrakul S., Rojanaporn D., Chaitankar V., Srimongkol A., Anurathapan U., Hongeng S., Kaewkhaw R. (2023). Ceftriaxone exerts antitumor effects in MYCN-driven retinoblastoma and neuroblastoma by targeting DDX3X for translation repression. Mol. Oncol..

[136] Halakos E.G., Connell A.J., Glazewski L., Wei S., Mason R.W. (2019). Bottom up proteomics reveals novel differentiation proteins in neuroblastoma cells treated with 13-cis retinoic acid. J. Proteom..

[137] Halakos E.G., Connell A.J., Glazewski L., Wei S., Mason R.W. (2021). Bottom up proteomics identifies neuronal differentiation pathway networks activated by cathepsin inhibition treatment in neuroblastoma cells that are enhanced by concurrent 13-cis retinoic acid treatment. J. Proteom..

[138] Shilpa G., Renjitha J., Saranga R., Sajin F.K., Nair M.S., Joy B., Sasidhar B.S., Priya S. (2017). Epoxyazadiradione Purified from the Azadirachta indica Seed Induced Mitochondrial Apoptosis and Inhibition of NFkappaB Nuclear Translocation in Human Cervical Cancer Cells. Phytother. Res..

[139] Stickland L.H. (1941). The inhibition of glucolysis by glyceraldehyde. Biochem. J..

[140] Sakamoto A., Prasad K.N. (1972). Effect of DL-glyceraldehyde on mouse neuroblastoma cells in culture. Cancer Res..

[141] Marengo B., Meta E., Brullo C., De Ciucis C., Colla R., Speciale A., Garbarino O., Bruno O., Domenicotti C. (2020). Biological evaluation of pyrazolyl-urea and dihydro-imidazo-pyrazolyl-urea derivatives as potential anti-angiogenetic agents in the treatment of neuroblastoma. Oncotarget.

[142] Morretta E., Sidibe A., Spallarossa A., Petrella A., Meta E., Bruno O., Monti M.C., Brullo C. (2021). Synthesis, functional proteomics and biological evaluation of new 5-pyrazolyl ureas as potential anti-angiogenic compounds. Eur. J. Med. Chem..

[143] Morretta E., Belvedere R., Petrella A., Spallarossa A., Rapetti F., Bruno O., Brullo C., Monti M.C. (2021). Novel insights on the molecular mechanism of action of the anti-angiogenic pyrazolyl-urea GeGe-3 by functional proteomics. Bioorg Chem..

[144] Sardana D., Zhu C., Zhang M., Gudivada R.C., Yang L., Jegga A.G. (2011). Drug repositioning for orphan diseases. Brief. Bioinform..

[145] Li X., Li H., Li S., Zhu F., Kim D.J., Xie H., Li Y., Nadas J., Oi N., Zykova T.A. (2012). Ceftriaxone, an FDA-approved cephalosporin antibiotic, suppresses lung cancer growth by targeting Aurora B. Carcinogenesis.

[146] Matthay K.K., Villablanca J.G., Seeger R.C., Stram D.O., Harris R.E., Ramsay N.K., Swift P., Shimada H., Black C.T., Brodeur G.M. (1999). Treatment of high-risk neuroblastoma with intensive chemotherapy, radiotherapy, autologous bone marrow transplantation, and 13-cis-retinoic acid. Children’s Cancer Group. N. Engl. J. Med..

[147] Neuman T., Stephens R.W., Salonen E.M., Timmusk T., Vaheri A. (1989). Induction of morphological differentiation of human neuroblastoma cells is accompanied by induction of tissue-type plasminogen activator. J. Neurosci. Res..

[148] Moreno L., Caron H., Geoerger B., Eggert A., Schleiermacher G., Brock P., Valteau-Couanet D., Chesler L., Schulte J.H., De Preter K. (2017). Accelerating drug development for neuroblastoma—New Drug Development Strategy: An Innovative Therapies for Children with Cancer, European Network for Cancer Research in Children and Adolescents and International Society of Paediatric Oncology Europe Neuroblastoma project. Expert Opin. Drug Discov..

[149] Johnsen J.I., Dyberg C., Fransson S., Wickstrom M. (2018). Molecular mechanisms and therapeutic targets in neuroblastoma. Pharmacol. Res..

[150] McKerrow J.H. (2018). Update on drug development targeting parasite cysteine proteases. PLoS Negl. Trop. Dis..

[151] Tucker E.R., Poon E., Chesler L. (2019). Targeting MYCN and ALK in resistant and relapsing neuroblastoma. Cancer Drug Resist..

[152] Wang Y., Chen K., Cai Y., Cai Y., Yuan X., Wang L., Wu Z., Wu Y. (2017). Annexin A2 could enhance multidrug resistance by regulating NF-kappaB signaling pathway in pediatric neuroblastoma. J. Exp. Clin. Cancer Res..

[153] Galenkamp K.M., Carriba P., Urresti J., Planells-Ferrer L., Coccia E., Lopez-Soriano J., Barneda-Zahonero B., Moubarak R.S., Segura M.F., Comella J.X. (2015). TNFalpha sensitizes neuroblastoma cells to FasL-, cisplatin- and etoposide-induced cell death by NF-kappaB-mediated expression of Fas. Mol. Cancer.

[154] Tang J., He J., Guo H., Lin H., Li M., Yang T., Wang H.Y., Li D., Liu J., Li L. (2023). PTBP2-Mediated Alternative Splicing of IRF9 Controls Tumor-Associated Monocyte/Macrophage Chemotaxis and Repolarization in Neuroblastoma Progression. Research.

[155] Levin V.A., Panchabhai S.C., Shen L., Kornblau S.M., Qiu Y., Baggerly K.A. (2010). Different changes in protein and phosphoprotein levels result from serum starvation of high-grade glioma and adenocarcinoma cell lines. J. Proteome Res..

[156] Chae S.Y., Nam D., Hyeon D.Y., Hong A., Lee T.D., Kim S., Im D., Hong J., Kang C., Lee J.W. (2021). DNA repair and cholesterol-mediated drug efflux induce dose-dependent chemoresistance in nutrient-deprived neuroblastoma cells. iScience.

[157] Yang C., Tan J., Zhu J., Wang S., Wei G. (2017). YAP promotes tumorigenesis and cisplatin resistance in neuroblastoma. Oncotarget.

[158] Merlos Rodrigo M.A., Buchtelova H., de Los Rios V., Casal J.I., Eckschlager T., Hrabeta J., Belhajova M., Heger Z., Adam V. (2019). Proteomic Signature of Neuroblastoma Cells UKF-NB-4 Reveals Key Role of Lysosomal Sequestration and the Proteasome Complex in Acquiring Chemoresistance to Cisplatin. J. Proteome Res..

[159] Marusyk A., Almendro V., Polyak K. (2012). Intra-tumour heterogeneity: A looking glass for cancer?. Nat. Rev. Cancer.

[160] McGranahan N., Swanton C. (2017). Clonal Heterogeneity and Tumor Evolution: Past, Present, and the Future. Cell.

[161] Louis C.U., Shohet J.M. (2015). Neuroblastoma: Molecular pathogenesis and therapy. Annu. Rev. Med..

[162] Acosta S., Lavarino C., Paris R., Garcia I., de Torres C., Rodriguez E., Beleta H., Mora J. (2009). Comprehensive characterization of neuroblastoma cell line subtypes reveals bilineage potential similar to neural crest stem cells. BMC Dev. Biol..

[163] Gartlgruber M., Sharma A.K., Quintero A., Dreidax D., Jansky S., Park Y.G., Kreth S., Meder J., Doncevic D., Saary P. (2021). Super enhancers define regulatory subtypes and cell identity in neuroblastoma. Nat. Cancer.

[164] Lopez-Carrasco A., Berbegall A.P., Martin-Vano S., Blanquer-Maceiras M., Castel V., Navarro S., Noguera R. (2021). Intra-Tumour Genetic Heterogeneity and Prognosis in High-Risk Neuroblastoma. Cancers.

[165] van Groningen T., Akogul N., Westerhout E.M., Chan A., Hasselt N.E., Zwijnenburg D.A., Broekmans M., Stroeken P., Haneveld F., Hooijer G.K.J. (2019). A NOTCH feed-forward loop drives reprogramming from adrenergic to mesenchymal state in neuroblastoma. Nat. Commun..

[166] Shi H., Tao T., Abraham B.J., Durbin A.D., Zimmerman M.W., Kadoch C., Look A.T. (2020). ARID1A loss in neuroblastoma promotes the adrenergic-to-mesenchymal transition by regulating enhancer-mediated gene expression. Sci. Adv..

[167] Gomez R.L., Ibragimova S., Ramachandran R., Philpott A., Ali F.R. (2022). Tumoral heterogeneity in neuroblastoma. Biochim. Biophys. Acta Rev. Cancer.

[168] Braekeveldt N., von Stedingk K., Fransson S., Martinez-Monleon A., Lindgren D., Axelson H., Levander F., Willforss J., Hansson K., Ora I. (2018). Patient-Derived Xenograft Models Reveal Intratumor Heterogeneity and Temporal Stability in Neuroblastoma. Cancer Res..

[169] Perkel J.M. (2021). Single-cell proteomics takes centre stage. Nature.

[170] Arias-Hidalgo C., Juanes-Velasco P., Landeira-Vinuela A., Garcia-Vaquero M.L., Montalvillo E., Gongora R., Hernandez A.P., Fuentes M. (2022). Single-Cell Proteomics: The Critical Role of Nanotechnology. Int. J. Mol. Sci..

[171] Lohani V., Akhiya A.R., Kundu S., Akhter M.Q., Bag S. (2023). Single-Cell Proteomics with Spatial Attributes: Tools and Techniques. ACS Omega.

[172] Torok S., Vegvari A., Rezeli M., Fehniger T.E., Tovari J., Paku S., Laszlo V., Hegedus B., Rozsas A., Dome B. (2015). Localization of sunitinib, its metabolites and its target receptors in tumour-bearing mice: A MALDI-MS imaging study. Br. J. Pharmacol..

[173] Ryu S., Hayashi M., Aikawa H., Okamoto I., Fujiwara Y., Hamada A. (2018). Heterogeneous distribution of alectinib in neuroblastoma xenografts revealed by matrix-assisted laser desorption ionization mass spectrometry imaging: A pilot study. Br. J. Pharmacol..

[174] Wu Z., Hundsdoerfer P., Schulte J.H., Astrahantseff K., Boral S., Schmelz K., Eggert A., Klein O. (2021). Discovery of Spatial Peptide Signatures for Neuroblastoma Risk Assessment by MALDI Mass Spectrometry Imaging. Cancers.

[175] Chen Y.N., LaMarche M.J., Chan H.M., Fekkes P., Garcia-Fortanet J., Acker M.G., Antonakos B., Chen C.H., Chen Z., Cooke V.G. (2016). Allosteric inhibition of SHP2 phosphatase inhibits cancers driven by receptor tyrosine kinases. Nature.

[176] Li Y., Dong J., Qin J.J. (2024). Small molecule inhibitors targeting heat shock protein 90: An updated review. Eur. J. Med. Chem..

[177] Regan P.L., Jacobs J., Wang G., Torres J., Edo R., Friedmann J., Tang X.X. (2011). Hsp90 inhibition increases p53 expression and destabilizes MYCN and MYC in neuroblastoma. Int. J. Oncol..

[178] Hsieh C.H., Huang C.T., Cheng Y.S., Hsu C.H., Hsu W.M., Chung Y.H., Liu Y.L., Yang T.S., Chien C.Y., Lee Y.H. (2023). Homoharringtonine as a PHGDH inhibitor: Unraveling metabolic dependencies and developing a potent therapeutic strategy for high-risk neuroblastoma. Biomed. Pharmacother..

[179] Saldivar J.C., Cortez D., Cimprich K.A. (2017). The essential kinase ATR: Ensuring faithful duplication of a challenging genome. Nat. Rev. Mol. Cell Biol..

[180] DeGregori J., Johnson D.G. (2006). Distinct and Overlapping Roles for E2F Family Members in Transcription, Proliferation and Apoptosis. Curr. Mol. Med..

[181] Ejeskar K., Krona C., Caren H., Zaibak F., Li L., Martinsson T., Ioannou P.A. (2005). Introduction of in vitro transcribed ENO1 mRNA into neuroblastoma cells induces cell death. BMC Cancer.

[182] Omenn G.S. (2006). Strategies for plasma proteomic profiling of cancers. Proteomics.

